# A Survey of Current Operations-Ready Thermospheric Density Models for Drag Modeling in LEO Operations

**DOI:** 10.1007/s40295-025-00558-8

**Published:** 2026-03-17

**Authors:** Shaylah Mutschler, Marcin Pilinski, Sean Bruinsma, Eric Sutton, W. Kent Tobiska, Delores Knipp, Tzu-Wei Fang, Steve Casali, Vishnuu Mallik, Brandon DiLorenzo, Christian Siemes

**Affiliations:** 1 Space Environment Technologies (SET), Street, Pacific Palisades, CA USA; 2 Laboratory for Atmospheric and Space Physics (LASP), University of Colorado at Boulder, Boulder, CO USA; 3 Space Geodesy Office, Centre National d’Etudes Spatiales (CNES), Toulouse, France; 4 Space Weather Technology, Research and Eductation Center (SWx TREC), University of Colorado at Boulder, Boulder, CO USA; 5 Smead Aerospace Engineering Sciences Department, University of Colorado at Boulder, Boulder, CO USA; 6 National Oceanic & Atmospheric Association (NOAA), Boulder, CO USA; 7 Omitron, Beltsville, MD USA; 8 Planet Labs PBC, San Francisco, CA USA; 9 Delft University of Technology, Delft, Netherlands

**Keywords:** Space weather, Satellite drag, Thermospheric density, LEO operations

## Abstract

In Low Earth Orbit (LEO), atmospheric drag is the largest contributor to trajectory prediction error. The current thermospheric density model used by the Combined Space Operations Center (CSpOC) in operations is the High Accuracy Satellite Drag Model (HASDM). Since HASDM is not available for use outside of the US Government, satellite operators are left to determine what publicly available, open-source density model they should integrate into their internal operational software. Given the ever more challenging nature of operations in LEO, it is imperative for satellite operators to update legacy density models to a state-of-the-art density model to provide improved trajectory predictions for collision risk assessment and vital day-to-day operational decisions. This article outlines four operations-ready thermospheric density models, describing their performance, computation time, required space weather inputs, and notes for implementation. Operations-ready models include the Drag Temperature Model (DTM), the Jacchia-Bowman 2008 (JB2008) model, the US Naval Research Laboratory Mass Spectrometer and Incoherent Scatter radar 2.0 (NRLMSIS 2.0) model, and the Thermosphere-Ionosphere-Electrodynamics General Circulation Model (TIE-GCM). US Government operational density models, HASDM and the Whole Atmosphere Model and Ionosphere Plasmasphere Electrodynamics (WAM-IPE) model, are included for comparison. Models are evaluated against *global* HASDM density and *local* GRACE-FO satellite accelerometer densities and Swarm mission densities. Additionally, comparisons between HASDM and WAM-IPE nowcast and forecast density are revealed for the first time publicly.

**Supplementary Information:**

The online version contains supplementary material available at 10.1007/s40295-025-00558-8.

## Introduction

The Low Earth Orbit (LEO) regime is becoming more congested as the number of satellites continues to grow with the establishment and rising popularity of SmallSat constellations. For example, SpaceX is in the process of creating a 12,000-satellite Starlink constellation in LEO, with more than 6000 SmallSats in orbit currently. The growth in the number of LEO objects directly increases the probability of unintentional collisions between objects due to accumulating space debris. This could lead to a runaway scenario known as the Kessler syndrome, where unavoidable cascading collisions occur, leading to a potentially unusable LEO orbital domain. To compound matters, this solar maximum, occurring between 2024–2027, is already higher than the previous solar maximum, thus causing larger perturbations due to drag from atmospheric density on LEO satellites. Given the ever more challenging nature of operations in LEO, it is imperative for satellite operators to update legacy density models to reduce density prediction errors and provide improved trajectory predictions for collision risk assessment and vital day-to-day operational decisions.

The Combined Space Operations Center (CSpOC) is responsible for the detection, identification, and daily tracking of all human-made objects in space. CSpOC also provides a conjunction analysis service that alerts satellite operators if their satellite exceeds a specific collision probability threshold with another Resident Space Object (RSO). The current thermospheric density model used by CSpOC in operations, the High Accuracy Satellite Drag Model (HASDM) [1], applies corrections to the Jacchia-Bowman 2008 (JB2008) model every 3 h using observations of 80+ calibration satellites. Since HASDM is not available for use outside of the US Government, satellite operators are left to determine which publicly available, open-source density model they should integrate into their internal operational software. This decision is nontrivial due to the number of available density models, each having variable performance dependent on several factors including space weather conditions and orbit altitude [2, 3].

This paper outlines several operations-ready thermospheric density models, describing their performance, computation time, required operational space weather input parameters, and notes for implementation. The goal is to provide satellite operators with information to help them determine which density model(s) may be most appropriate for their specific operational needs. *We define an operations-ready density model as a model that is well-documented, has verified and quantified model performance, and provides publicly available model code for implementation on a user’s own system*. An operations-ready density model must also be able to run in near real-time such that the processing speed is faster than the wall clock using reasonable computational hardware. The models that meet these criteria are the Drag Temperature Model (DTM), the JB2008 model, the US Naval Research Laboratory Mass Spectrometer and Incoherent Scatter radar 2.0 (NRLMSIS 2.0, shortened to MSIS2.0) model, and the Thermosphere–Ionosphere–Electrodynamics General Circulation Model (TIE-GCM). US Government operational density models, HASDM and the Whole Atmosphere Model and Ionosphere Plasmasphere Electrodynamics (WAM-IPE), are included for comparison in the Analysis section. WAM-IPE is run operationally at National Oceanic and Atmospheric Administration (NOAA) and was recently designated as one of the density models in the Department of Commerce’s (DoC) new Traffic Coordination System for Space (TraCSS), which is currently in development. All models included in this paper are at a Technology Readiness Level (TRL) of eight or nine, meaning that their performance has been validated under operational conditions or that they are currently run on an operational system.

This paper is organized as follows. Section 2 provides an overview of space weather, with subsections on the mechanisms that drive variations in thermospheric density, how we quantify space weather activity to drive density models, and an overview of thermospheric density. A description of satellite drag and its calculation is provided in Sect. 3. Section 4 reviews the various operations-ready and US government operational thermospheric density models. In Sect. 5 we analyze the performance of the operations-ready density models, comparing them to each other, the Gravity Recovery And Climate Experiment Follow-On (GRACE-FO) accelerometer-derived density, Swarm GPS-derived density, HASDM, and WAM-IPE. Additionally, comparisons between HASDM and WAM-IPE nowcast and *forecast* density are revealed for the first time publicly in Sect. 5. We conclude with a summary of our findings.

This paper is not meant to be a comprehensive evaluation of each density model, but rather an overview of the density modeling options available to LEO operators. The information provided is intended to assist satellite operators with updating their density models to meet the performance needs of an increasingly congested LEO environment and the current solar maximum conditions.

## Space Weather

### Sun-Earth Interaction

Disturbances in the near-Earth space environment are driven by the Sun and its interactions with geospace. The primary energy input into the thermosphere is solar irradiance in the form of soft X-ray Ultra-Violet (XUV, 1–30 nm), Extreme Ultra-Violet (EUV, 30–120 nm), Lyman-$\alpha$ (121 nm), and Far Ultra-Violet (FUV, 122–200 nm) [4]. Variations in XUV, EUV, Lyman-$\alpha$, and FUV solar irradiance drive variations in the overall heating of the thermosphere on a slower time scale than geomagnetic activity.

The second most important energy input to the thermosphere is geomagnetic activity caused by the interaction between the solar wind and the Earth’s magnetic field. More specifically, Coronal Mass Ejections (CME) and high-speed solar wind cause disturbances in the Earth’s magnetic field that lead to thermospheric density enhancements. CMEs are streams of plasma that erupt from the Sun and travel outward through the interplanetary medium. Depending on their polarity and direction of travel, some CMEs collide with Earth’s magnetic field and cause weak to strong geomagnetic storms depending on their polarity. The solar wind is an ongoing outflow of the Sun’s coronal plasma into the interplanetary medium. High-Speed Streams (HSS) emanate from open magnetic field regions in the Sun’s corona. Interactions between slow and fast solar wind cause Co-rotating Interaction Regions (CIR). When solar wind HSS and/or CIR interact with Earth, the field irregularities in the plasma may contribute to moderate geomagnetic storms. Both CMEs and high-speed solar wind can cause geomagnetic storms, which lead to thermospheric density enhancements. Knipp [5] provides a comprehensive text on these topics.

**Fig. 1:**
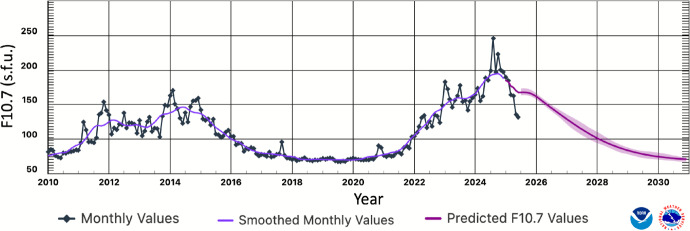
Solar cycle F10.7 cm radio flux progression (NOAA SWPC, 2025, https://www.swpc.noaa.gov/products/solar-cycle-progression)

The frequency and magnitude of thermospheric density enhancements stemming from solar short-wave irradiance (XUV, EUV, Lyman-$\alpha$, and FUV) variations and geomagnetic activity are dependent on the approximately 11 year solar cycle and 27 day solar rotation. During an 11 year solar cycle, the Sun experiences a solar minimum and maximum in the number of sunspots and active regions. We are currently experiencing the near-maximum of solar cycle 25. The Sun’s short-wave irradiance increases as it approaches solar maximum because active regions of the Sun (i.e., magnetically induced enhancements to the solar atmosphere above sunspots) produce the largest quantity of solar flux. Similarly, the frequency and magnitude of CMEs and solar flares peak during solar maximum or shortly thereafter. Figure 1 shows solar radio flux emissions at 10.7 cm (measured in solar flux units, or s.f.u., equivalent to $10^{-22}\,\mathrm {W\,m^{-2}\,Hz^{-1}}$) for solar cycles 24 and 25. The solar short-wavelength flux, represented by the F10.7 proxy, during this solar maximum is higher than what was predicted for this cycle and has already surpassed the peak F10.7 of the previous solar cycle.

### Solar and Geomagnetic Model Drivers

Solar and geomagnetic drivers play a crucial role as inputs in thermospheric density models. Kp, Ap, and F10.7 are commonly used legacy model drivers; additional, more contemporary drivers include Dst, S10, M10, Y10 and F30. This section provides a description of the operational solar and geomagnetic drivers utilized as inputs for thermospheric density models. A more comprehensive description of drivers can be found in the ISO International Standard on Earth upper atmosphere [6].

It is important to recognize a fundamental distinction of thermospheric density model drivers: they are categorized as either indices, proxies, or both. A solar irradiance proxy is a measurement of the solar flux at a particular wavelength but is used as a proxy for another wavelength because of its high correlation to the other wavelength. An example of this is F10.7, which serves as both a proxy for solar EUV emissions *and* an index of solar radio flux. F10.7 is an EUV proxy because it measures the solar radio flux at 10.7 cm wavelength (radio spectrum), which is not absorbed by the atmosphere and therefore has no direct impact on thermospheric density and can be measured from the ground regardless of terrestrial weather conditions.

**Fig. 2:**
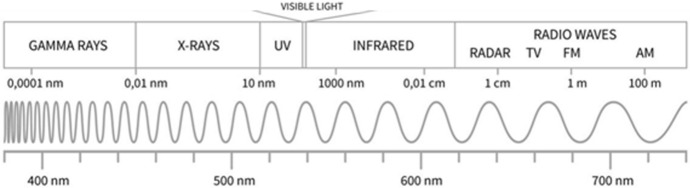
Solar irradiance wavelength spectrum. The visible portion of the spectrum is expanded at the bottom

The F10.7 is a very reliable and well-calibrated solar radio flux index at 10.7 cm, and it remains the most widely used EUV proxy for thermospheric density models, despite the availability of direct EUV measurements from space. Since 2013, the F30 proxy (solar radio flux at 30 cm), which mimics the EUV irradiance better than F10.7, has been used in some DTM versions. The M10 proxy is derived from the Mg II core-to-wing ratio [7] and is measured from the Geostationary Operational Environmental Satellite (GOES) series satellites via Extreme Ultraviolet and X-ray Sensors (EXIS)/Extreme Ultraviolet Spectrometer (EUVS) (Fig. 2). M10 indicates the heating of O_2_ occurring in Earth’s atmosphere at 100–110 km altitude by photons originating from the solar chromosphere and photosphere in the Schumann–Runge Continuum near 160 nm [8].

Thermospheric heating is predominantly driven by solar chromospheric EUV energy, complemented by contributions from coronal soft X-ray, Lyman-$\alpha$, and photospheric FUV wavelengths [9]. A range of solar irradiances in a variety of wavelengths emanate from the Sun. Upon reaching Earth, the Sun’s photons are absorbed by distinct atomic and molecular species, each at specific unit optical depth layers in the atmosphere [9]. It is with this understanding that Tobiska developed the M10 *proxy*, along with the S10 and Y10 *indices* [8] for utilization in the JB2008 model.

Each of these indices indicates the amount of solar flux energy being transferred to a particular altitude range of the Earth’s atmosphere. The S10 and Y10 indices are measurements of solar short-wave irradiance, which are absorbed by the atmosphere and therefore directly affect thermospheric density. The S10 index is the integrated 26–34 nm solar irradiance (EUV) that was originally measured by the Solar Extreme-ultraviolet Monitor (SEM) instrument on a number of satellites including the NASA/European Space Agency (ESA) Solar and Heliospheric Observatory (SOHO) research satellite (Lagrange Point 1 (L1) on the Earth-Sun line) [8] and is now operationally observed by the GOES series satellites. S10 represents the heating of atomic Oxygen occurring above 200 km altitude in Earth’s atmosphere by photons originating from the solar chromosphere. The Y10 index is a combination of 0.1$-$0.8 nm X-rays and 121 nm Lyman-$\alpha$; it is weighted to represent mostly X-rays during solar maximum and mostly Lyman-$\alpha$ during moderate and low solar activity [8]. Measurements for the Y10 index are collected via the X-ray spectrometer (XRS) and EXIS instruments on NOAA GOES satellites. Y10 represents the heating of N_2_, O_2_, H_2_O, nitric oxide (NO) occurring in Earth’s atmosphere at 85–90 km altitude by photons originating from the solar transition region and corona. F10.7, M10, S10, and Y10 are all daily indices and are provided in solar flux units (s.f.u.).

The Kp/Ap, and Dst indices describe geomagnetic activity. They are proxies for upper atmosphere heating due to interaction with the solar wind. Kp/Ap characterizes the intensity of the geomagnetic field disturbance derived from 13 magnetometers distributed across mid-latitudes worldwide.^1^ The Ap index is the linear form of the quasi-logarithmic Kp index; a mapping between the two indices is provided by NOAA.^2^ Kp ranges from 0 to a maximum of 9 (dimensionless), while Ap ranges from 0 to 400 (units of 2 nT). Both have a time resolution of 3 h. Dst is an indicator of the strength of the storm-time ring current in the inner magnetosphere and is calculated hourly using measurements from four off-equatorial magnetic observatories^3^ [10]. During the main phase of geomagnetic storms, the ring current becomes highly energized and produces a southward-directed magnetic field perturbation at low latitudes on the Earth’s surface [11]. This is opposite to the normal northward-directed main field. Dst has a time resolution of 1 h, which allows for a more temporally resolved assessment of geomagnetic storm evolution compared to the traditional 3 h cadence of Kp and Ap indices. While this difference primarily reflects the cadence at which these indices are computed and disseminated, rather than a fundamental limitation, the hourly resolution of Dst makes it more suitable for studies requiring finer temporal detail. However, higher-cadence versions of Kp (or Ap) indices, Hp30 and Hp60 at 30 and 60 min cadence [12], respectively, are presently operationally available.^4^

**Fig. 3:**
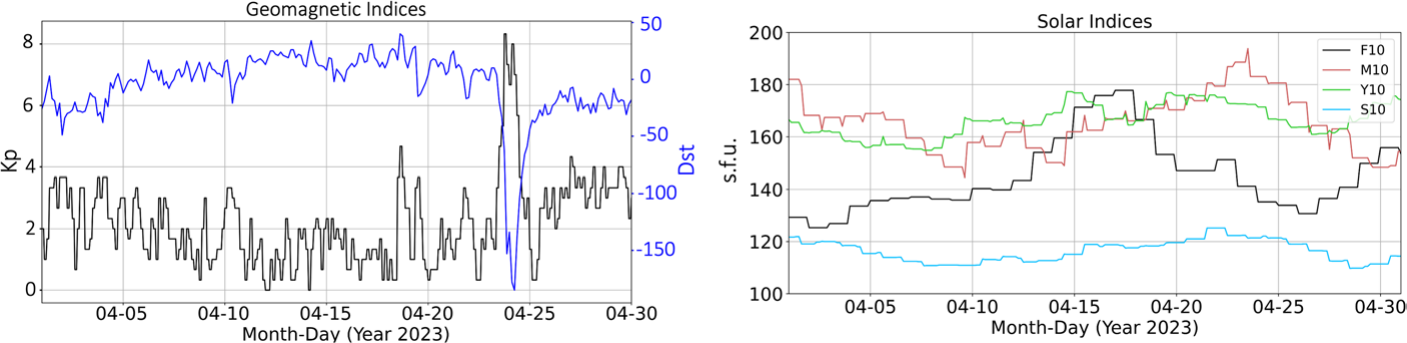
April 2023 solar and geomagnetic drivers

Dst has a time resolution of 1 h, which provides a finer assessment temporally of the evolution of geomagnetic storms compared to Ap or Kp. Figure 3 provides plots of the Kp, Dst, F10, M10, S10, and Y10 drivers during the month of April 2023.

Improved thermospheric density forecasts are a critical need identified by the Space Weather Operations, Research, and Mitigation (SWORM) Working Group, a Federal interagency coordinating body [13]. Errors in thermospheric density predictions are a function of both the density model and the model drivers. The performance of both components is influenced by space weather conditions, particularly geomagnetic activity, with driver predictions degrading rapidly as the forecast horizon increases. This paper primarily explores performance based on density model rather than model drivers and their predictions. Although several sources provide forecast drivers, the accuracy of these forecasts varies depending on several factors. In a recent study benchmarking forecast drivers and their success, Licata et al. [14] found that the performance of forecast JB2008 indices degrades during periods of high activity levels, when the Sun is more volatile. They concluded that flaring active region evolution is still poorly predicted, affecting the S10 index, while the magnitude and timing for the Dst index are also not well predicted.

The various solar and geomagnetic drivers that are required for each operations-ready density model are provided in Table 1. Operational solar and geomagnetic drivers are available from a number of sources. The GFZ German Research Centre for Geosciences provides Kp, Ap, and F10.7 nowcasts.^5^ NOAA Space Weather Prediction Center (SWPC) provides a 3 day Kp forecast with a 3 h time resolution,^6^ as well as 45 day Kp and F10.7 forecasts with a 1 day time resolution.^7^ CelesTrak provides Kp, Ap, and F10.7 nowcasts, 3 day Kp forecasts (at a 3 h time resolution), and a 45 day forecast of the daily Kp, Ap, and F10.7.^8^ The forecast drivers provided by CelesTrak are the same as those provided by NOAA SWPC. The quick-look Dst index is released through the World Data Center in Kyoto, Japan.^9^ Collecte Localisation Satellites (CLS) provides the F30 nowcast and 30 day forecasts.^10^ SET provides operational F10.7, M10, S10, Y10, Ap, and Dst indices, updated hourly, via a subscription service on the U.S. Space Force Unified Data Library (UDL). The SET UDL subscription service includes nowcasts and a 6 day forecast with a 3 h time granularity of all solar indices. The 3 day Ap forecast is derived from the NOAA forecast. The SET predictions of F10.7, S10, M10, Y10 are produced independently using a linear predictive algorithm that incorporates persistence and recurrence; persistence is weighted for recent epochs and recurrence picks up the 27 day solar rotation variation [8]. However, only up to 4 month predictions are available via subscription.

**Table 1:** Required space weather drivers for each operations-ready density model

Model / driver	Kp/Ap	Dst	F10.7	S10, M10, Y10
DTM2020	X		X	
MSIS2.0	X		X	
JB2008	X	X	X	X, X, X
TIE-GCM	X		X	

### Thermospheric Density

Thermospheric density is highly dynamic and its variability depends on a number of factors, including solar activity, geomagnetic activity, diurnal cycle (local time), season, altitude, and latitude. Most of these factors are directly linked to the Sun. Solar cycle and solar activity describe the Sun’s short-wave irradiance, and geomagnetic activity is a measure of disturbance in the Earth’s magnetic field caused by solar wind (i.e., particles) and interplanetary magnetic field (IMF). The Sun-Earth geometry leads to a seasonal, diurnal, and semidiurnal cycle. Because thermospheric density decreases exponentially with altitude, its impact on orbital trajectories is limited primarily to LEO.

Periods of time when there is little to no geomagnetic field disturbance and relatively low solar flux are referred to as quiet conditions; whereas storm-time refers to periods of time when there is a major disturbance in the near-Earth space environment caused by a geomagnetic storm or solar activity. Density is typically highest around 2–4 pm local time, and lowest from 2 to 5 am local time and in the polar region experiencing winter below 500–600 km; at higher altitudes, the so-called winter Helium bulge causes the maximum to sit over the winter hemisphere [15].

## Satellite Drag

An object in LEO experiences atmospheric drag caused by particles in the atmosphere colliding with the surface of the object. In fact, atmospheric drag is a significant perturbation on LEO object motion, causing the largest uncertainty for orbit predictions. Drag acts primarily in the opposite direction of the object’s velocity vector and effectively dissipates orbit energy. A simplifying assumption of aerodynamic symmetry is often made that results in the drag force that is directed opposite the relative velocity. The magnitude of the force due to drag is directly dependent on neutral density. The acceleration due to drag is generally modeled as follows:

1 $$\begin{aligned} \overrightarrow{a}_\textrm{drag} = \ - \frac{1}{2}\rho \frac{C_{D}A}{m}v_\textrm{rel}^{2}\frac{{\overrightarrow{v}}_\textrm{rel}}{\left| {\overrightarrow{v}}_\textrm{rel} \right| } \end{aligned}$$

where,

2 $$\begin{aligned} {\overrightarrow{v}}_\textrm{rel} = \ \frac{d\overrightarrow{r}}{dt} - {\overrightarrow{\omega }}_{\oplus } \times \overrightarrow{r} \end{aligned}$$

is the velocity vector relative to an atmosphere co-rotating with the Earth; C_D_ is the drag coefficient; A is the exposed cross-sectional area to the particle flow (area projected onto a plane normal to ${\overrightarrow{\textbf{v}}}_{\textbf{rel}}$); m is the object’s mass; ${\overrightarrow{\mathbf {\omega }}}_{\mathbf {\oplus }}$ is Earth’s angular velocity; $\overrightarrow{\textbf{r}}$ is the object position vector in an Earth Centered Inertial reference frame; and $\rho$ is thermospheric density [16]. The drag coefficient, mass, and area of the object are typically combined to form the ballistic coefficient ($\mathbf {\beta =}$ C_D_A/m). C_D_ and A can vary in time as a function of spacecraft attitude. The cross-sectional area A is computed using a spacecraft geometry model (sometimes referred to as a macro model) and attitude information. There are several options for obtaining the C_D_ of a satellite, including a) estimating C_D_ by fitting observations of the drag and the chosen density model, and b) computing the physical C_D_ through a variety of methods. A more detailed overview of these methods can be found in Thayer et al. [9]. Estimating C_D_ inherently de-biases the density model and is a popular choice in space operations. The physical C_D_ is a function of ambient conditions such as atmospheric gas temperature, velocity, and composition. There is presently no consensus on which C_D_ model is best, but the more realistic ones require the individual species densities, which can be provided by models such as MSIS or DTM. An example of a physical C_D_ model is posted here.^11^ More information on C_D_ can be found in the following publications [17–22]. Additionally, geomagnetic activity disturbances have been shown to produce enhanced neutral winds in the upper atmosphere. The speed of the neutral winds can, at times, approach 1 km/s, which can cause rapid unpredictable fluctuations in apparent velocity for a LEO satellite [23].

## Thermospheric Density Models

Current methods that model thermospheric density can be categorized as empirical or physics-based. Empirical models use both short and long-term trends from measurements using multiple instruments to provide a parameterized mathematical formulation of the system. Physics-based models represent the underlying dynamics of the system but require substantially more computing resources than empirical models [24]. Although empirical models have historically been applied in operations due to their faster and more efficient nature, as computation power becomes more accessible, the incorporation of physics-based models, like the TIE-GCM is becoming tractable in operations.

Another type of density modeling is data assimilative, where direct or indirect density measurements are assimilated to apply corrections to an empirical or physics-based background density model. HASDM is an example of a data assimilative density model that uses an empirical background model, JB2008, and assimilates indirect measurements of density in the form of satellite arcs [1, 25, 26]. Current research efforts are exploring data assimilation with both empirical and physics-based models. For example, Dragster assimilates orbital observations to estimate drivers of empirical density models [27], and both the Iterative Driver Estimation and Assimilation (IDEA) [28] and SoleiTool [29] assimilate high-cadence accelerometer data to estimate drivers of a physics-based density model. Nevertheless, other than HASDM, none of the data assimilation density models are operational at this time.

### Operations-Ready Density Models

This section provides an overview of the operations-ready density models. These are models that are well-documented, model performance has been verified and quantified, and the model code is publicly available for implementation on a user’s system. For each model, we provide information such as model inputs/outputs, where to obtain the model and required inputs, general model performance, and model limitations.

#### DTM

**Fig. 4:**
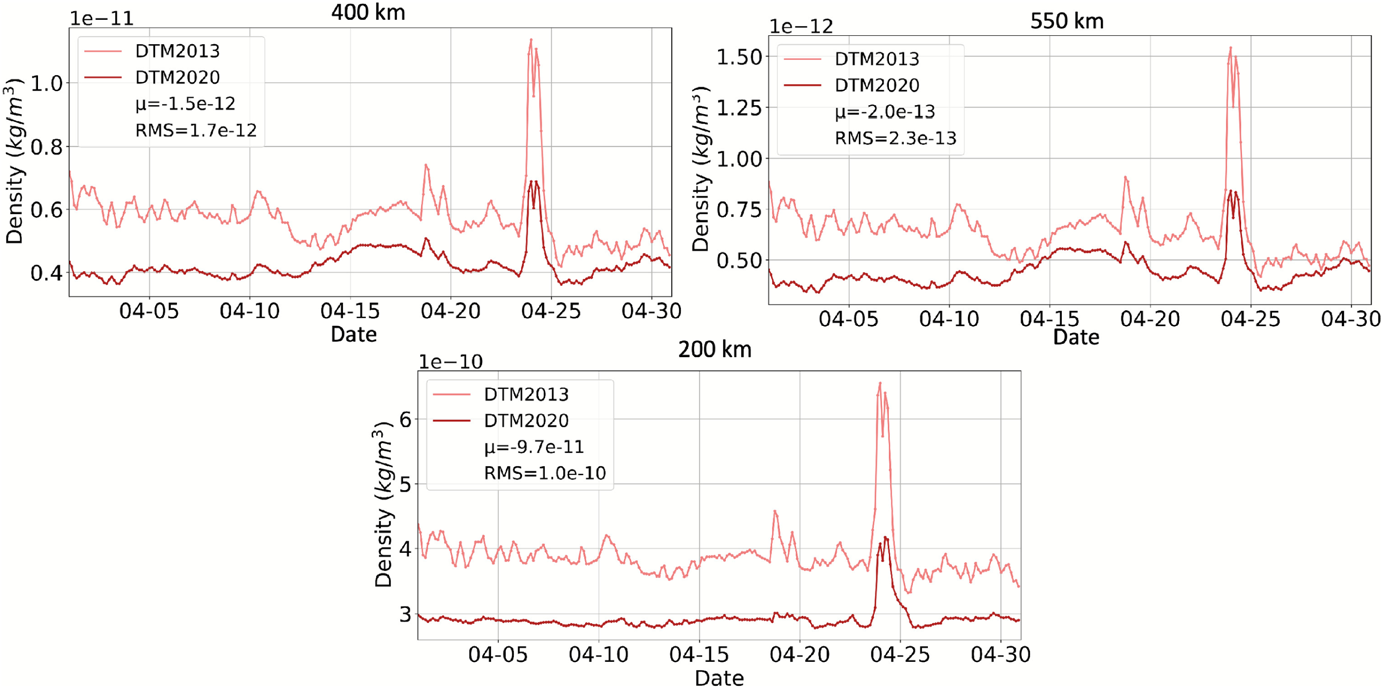
April 2023 comparison of DTM2020 and DTM2013 global mean density. Mean and RMS differences are shown in the legend

The first Drag Temperature Model, DTM78 [30], was developed in the seventies. The latest release at the time of writing is DTM2020 [31]. DTM is an empirical model and requires a license for operational use. It was constructed by optimally fitting model coefficients to the complete CHAllenging Mini satellite Payload (CHAMP) [32], GRACE [33] and Gravity field and steady-state Ocean Circulation Explorer (GOCE) [34] high-resolution accelerometer-inferred density datasets, as well as Swarm A densities [35], Stella daily-mean densities [33], and daily-mean global average mass densities at 250 and 325 km altitude derived from two-line orbital element sets (TLE) [36]. A key difference between the operational version of DTM2020 and DTM2013 is the choice of model drivers; the operational version of DTM2020 is driven by F10.7 and Kp, while DTM2013 uses F30 and Kp. Secondly, the density datasets were not adjusted to the U.S. Space Force HASDM model [1] by means of scaling factors, as described for GOCE data in [34]. Instead, CHAMP, GOCE, and Swarm A densities were considered of higher absolute accuracy and used unmodified. Consequently, DTM2020 densities are on average 20–30% smaller than DTM2013 and HASDM. Smaller absolute densities are consistent with MSIS2.0 [37], which also predicts smaller densities than NRLMSISE-00 (shortened to MSIS-00). Figure 4 provides a comparison between DTM2020 and DTM2013 global mean density at 200, 400, and 550 km altitude for the month of April 2023. The mean and Root Mean Square (RMS) differences between DTM2013 and DTM2020 over the month are included in the plot legend. DTM2020 density is noticeably lower than DTM2013 during both the quiet and storm conditions during this time period.

In DTM2020, total density is calculated via the addition of the partial densities of the main thermosphere constituents (N_2_, O_2_, O, He, H), under the hypothesis of independent static diffuse equilibrium. Temperatures and density variations as a function of latitude, local solar time, season, solar flux, and geomagnetic activity are modeled by means of a spherical harmonic function. DTM2020 ranges from 120 to 1500 km altitude. DTM2020 is less precise for high solar activity (81 day mean F10.7 > 180) due to the lack of representation of those conditions in the density datasets used to build the model. DTM2020 overall performance degrades at higher altitudes (>500 km). However, because DTM models constituents, helium modeling is qualitatively correct. As a result, it captures the winter helium bulge, an enhancement in helium concentration in the winter hemisphere of the thermosphere by 1–2 orders of magnitude relative to the summer hemisphere.

DTM is currently run at the National Centre for Space Studies (CNES) (all DTM versions), ESA, the United Kingdom (UK) Met Office (DTM2013), and NASA Community Coordinated Modeling Center (CCMC) (DTM 2013 and 2020). DTM is also integrated into Bernese GNSS software (DTM 2013 and 2020) [38], NASA Jet Propulsion Lab’s (JPL) Mission Analysis, Operations, and Navigation Toolkit Environment (MONTE) (DTM 2013 and 2020) (NASA JPL, 2016), NASA JPL’s GNSS-Inferred Positioning System (Gipsy-X) (DTM2013) [39], NASA Goddard’s GEODYN (DTM 94 and 2013),^12^ the Orekit astrodynamics toolkit (DTM2000),^13^ and the Orbit Determination Tool Kit (ODTK) (DTM 2013 and 2020).^14^ DTM2020 Fortran code (with Python wrapper option) and documentation are available.^15^ Running on a standard modern laptop, DTM takes seconds to produce 1 day of global density with a 3 h time resolution and a spatial resolution of 10-km altitude, 10-deg latitude, and 1 h local time.

#### JB2008

JB2008 is an empirical thermospheric density model and serves as the background model for HASDM, the operational thermospheric density model used by the Space Force for Space Traffic Management (STM). It is the most recent model in the line of Jacchia and Jacchia-Bowman models beginning with the Jacchia Reference Atmosphere in 1970 [40]. JB2008 is the recommended model internationally [6] for specifying neutral densities in LEO as they affect satellite drag. Four different density data sources were used in the development of the JB2008 model. These sources included Air Force daily density values from 1997 to 2007, Air Force HASDM densities from 2001 to 2005, CHAMP accelerometer densities from 2001 to 2005, and GRACE accelerometer densities from 2002 to 2005. In addition to F10.7, JB2008 uses solar irradiance indices M10, S10, and Y10 that are derived from solar short-wave irradiance space-based sensor data. It was the ability to insert more physics into thermospheric density drivers (S10, M10, Y10) in the form of specific solar wavelengths linked to optical depths that enabled JB2008 to substantially reduce density uncertainties [11, 8, 41]. The Dst geomagnetic index is used during storm conditions, while the Ap geomagnetic index is applied during quiet to slightly disturbed non-storm conditions. Use of the Dst index during storm conditions instead of the Ap index greatly reduced density errors, especially during major geomagnetic storm periods. The model solves for exospheric temperature and semiannual density corrections to obtain global thermospheric density variations. The JB2008 model was validated through comparisons of accurate daily density drag data previously computed for numerous satellites at altitudes of 200–1100 km, and from precise CHAMP and GRACE satellite accelerometer density data.

JB2008 does not model individual species densities, and therefore experiences performance degradation at altitudes higher than the oxygen-helium transition layer (400-700 km altitude depending on solar activity) where helium becomes the dominant species. This impacts modeling of phenomena such as the winter helium bulge. JB2008 also does not capture post-storm cooling, which is where post-storm density decays to lower than pre-storm levels due to overproduction of NO [42]; this is an area of active research in the thermosphere modeling community.

JB2008 is driven by recent, current, and predicted solar and geomagnetic indices provided by SET, including the S10, M10, Y10, and F10 solar indices as well as Dst and Ap geomagnetic indices [8]. Model Fortran code, Python wrapper, and historical model inputs are available.^16^ JB2008 is also included in the Orekit astrodynamics toolkit^17^ and MATLAB,^18^ and is run at CCMC. Running on a standard modern laptop, JB2008 takes seconds to produce 1 day of global density with a 3 h time resolution and spatial resolution of 10-deg latitude, 10-deg longitude, and 10-km altitude ranging 120 to 1500 km.

#### MSIS

**Fig. 5:**
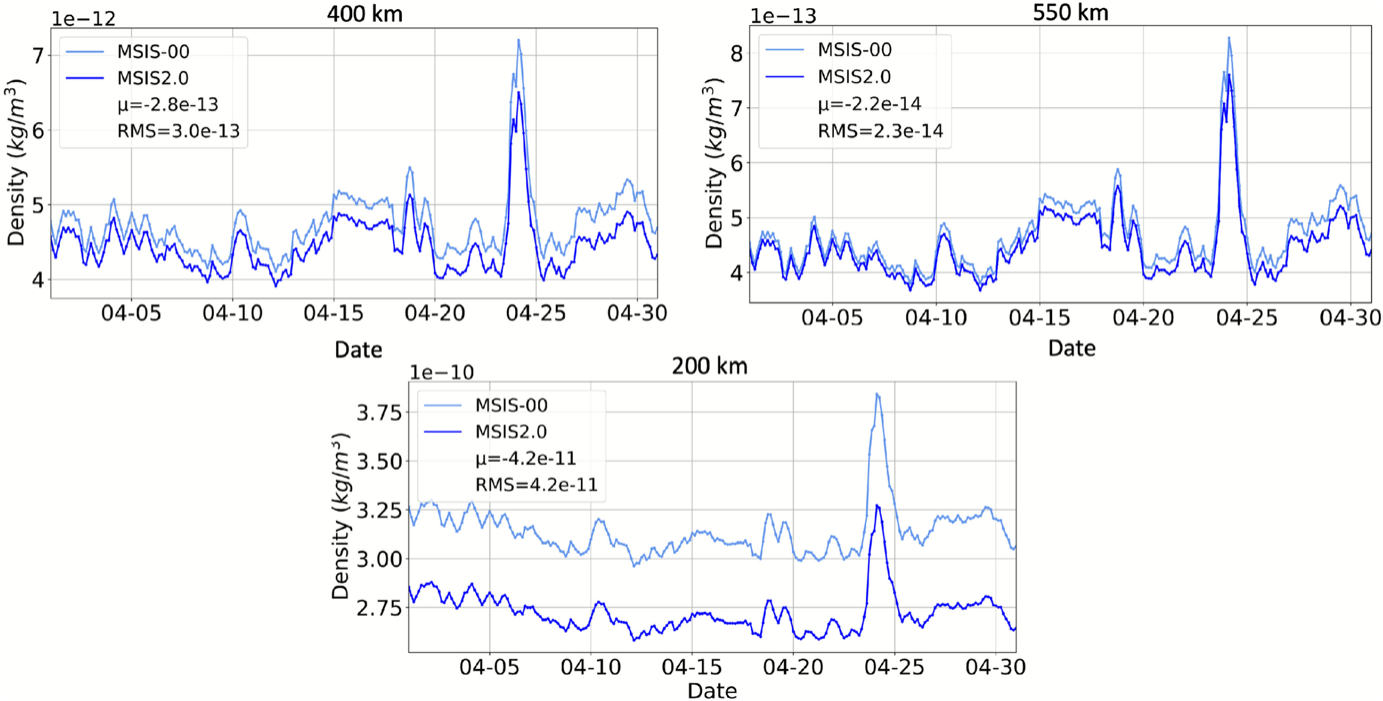
April 2023 comparison of MSIS2.0 and MSIS-00 global mean density. Mean and RMS differences are shown in the legend

The ISO International Standard 14222 on Earth’s upper atmosphere recommends using the MSIS2.0 empirical model for relative constituent abundances. The MSIS series of models provide atmospheric temperature, number densities of eight species, and mass density. MSIS2.0 was developed in 2020 as an improvement to MSIS-00. There were several updates implemented to create MSIS2.0, including the incorporation of extensive new (post-2000) lower and middle atmosphere temperature, O, and H data, along with global average thermospheric mass density derived from satellite orbits [37]. Additionally, major changes to the model formulation were implemented, including a temperature-dependent connection between densities in the lower/middle atmosphere and the thermosphere [37]. As a result of all the updates, N_2_ and O densities (the major constituents below 400 km altitude) in the thermosphere are lower in MSIS2.0 than MSIS-00. Figure 5 provides a comparison between MSIS2.0 and MSIS-00 global mean density at 200, 400, and 550 km altitude for the month of April 2023. The difference between MSIS2.0 and MSIS-00 is largest, relatively, at 200 km altitude with approximately a 10% difference, and lowest at 550 km altitude with a difference of only a few percentage points. The mean and RMS differences between MSIS2.0 and MSIS-00 over the month are included in the plot legend.

The MSIS2.0 model generates a density value when provided with local time, altitude, day of year (DOY), F10.7 proxy, and Ap. Similar to DTM, MSIS2.0 requires a license for operational use. Fortran code and data samples are available.^19^ A python wrapper of MSIS-00 and MSIS2.0 is available.^20^ MSIS-00 is available as part of Orekit.^21^ Both MSIS2.0 and MSIS-00 are run at NASA CCMC. A version of MSIS is integrated into several software packages, including NASA JPL’s Gipsy-X, NASA Goddard’s GEODYN,^22^ and NASA’s General Mission Analysis Tool (GMAT).^23^ Running on a standard modern laptop, MSIS2.0 takes seconds to produce 1 day of global density with a 3 h time resolution and spatial resolution of 10-deg latitude, 10-deg longitude, and 10 km ranging 120–1500 km altitude.

#### TIE-GCM

TIE-GCM is a physics-based space weather model developed at the National Center for Atmospheric Research (NCAR) High-Altitude Observatory (HAO) [43]. The original version of the model, the thermosphere general circulation model (TGCM), was developed by Dickinson et al. [44, 45] and Roble et al. [46]. In its current form, TIE-GCM is a global 3D numerical model that simulates the coupled thermosphere-ionosphere system from about 97 to 500–600 km altitude, the latter being dependent on solar activity [47]. For higher altitudes, an extrapolation scheme must be included for each neutral species based on its exospheric scale height. TIE-GCM self-consistently solves the fully coupled, nonlinear, hydrodynamic, thermodynamic, and continuity equations of the neutral gas, the ion and electron energy and momentum equations, the ion continuity equation, and neutral wind dynamo. The standard spatial resolution is a 5-deg by 5-deg horizontal grid although higher resolution runs can be configured. A pressure-based vertical coordinate is used and must be converted to altitude before computing drag. Note that with the lower boundary set at around 100 km, TIE-GCM does not realistically capture the thermosphere changes associated with perturbations from the lower atmosphere.

TIE-GCM can be driven by several methods and data streams but in its default setting is primarily driven by forcing parameters, Kp and F10.7; however, several additional input parameters and model tuning parameters can be provided as model input. More specifically, solar inputs are driven using the F10.7 proxy [43]. Tidal forcing at the lower boundary is specified by the Global Scale Wave Model. While running in standard resolution, semi-annual and annual density periodicities are taken into account by applying seasonal variation of the eddy diffusivity coefficient at the lower boundary [43, 48]. The electrodynamic potential field is internally generated at middle and low latitudes using the model densities and neutral winds. This is merged with a magnetospheric potential at high latitudes, using one of two available empirically driven models: (1) the empirical formulation of the Heelis high-latitude ionospheric convection model, which is driven by Kp, and is the standard TIE-GCM input [49], (2) the Weimer empirical model, which uses upstream solar wind and IMF as input [50]. Recent developments to TIE-GCM (TIE-GCM 2.0 released in 2015) include the addition of helium for high-altitude extension and lower boundary options [47]. This is important for representing thermospheric mass density above 400–500 km where helium becomes the dominant species. Species densities for O, O_2_, N_2_, N, NO, and helium (HE) are available as output in TIE-GCM.

Validation was performed via comparison to CHAMP, GRACE and GOCE density data during 13 geomagnetic storms [51]. Model density data from several models (MSIS-00, JB2008, DTM2013, the Coupled Thermosphere Ionosphere Plasmasphere Electrodynamics Model (CTIPe), and TIE-GCM) were compared to the in-situ satellite density measurements and it was found that TIE-GCM and DTM2013 had the best performance [51]. More thorough model assessment is still required to evaluate dependencies on solar cycle, local time, and altitude effects under storm conditions.

Similar to DTM and MSIS2.0, TIE-GCM 2.0 requires a license for operational use; earlier versions of TIE-GCM do not have this restriction. Model code and documentation are available.^24^ TIE-GCM is also available to run via Docker,^25^ provided by Space Weather Technology, Research and Education Center (SWx TREC) at the University of Colorado Boulder (CUB). TIE-GCM is run at NASA CCMC. Running on a standard modern laptop, TIE-GCM takes less than 10 min to produce 1 day of global density with a 1 h time resolution and spatial resolution of 5-deg latitude, 5-deg longitude, and vertical pressure level size of a half-scale height ranging from approximately 100–600 km altitude. For context, typical thermosphere scale heights in the 300–600 km region range from about 25 to 75 km [52].

### US Government Operations Density Models

This section provides a description of two US Government operational ready density models, HASDM and WAM-IPE. These models are included for comparison in the Analysis section.

#### US Space Force HASDM

HASDM is the operational density model used by the US Space Force CSpOC. It assimilates ground-based radar measurements of 80+ calibration satellites to solve for temperature corrections, calibrating the JB2008 density model and producing a global thermospheric density solution [1, 25, 26]. Intensive sensor tasking is made available for this effort, which allows for the collection of approximately 500 measurements per day per calibration satellite. The adjusted atmosphere is forecast beyond the current epoch using JB2008 forecast indices out to 144 h (6 days) for STM.

In 2020, Space Environment Technologies released a publicly available HASDM density database spanning 2000 to 2019.^26^ While real-time HASDM operational densities remain restricted to the U.S. Government, the historical operational nowcast densities spanning two solar cycles are accessible via the SET website.^27^ An extension of the database through mid-2025 is expected to be publicly released early 2026. The April 2023 HASDM densities used in this study are part of that upcoming release and are not yet publicly available. There is a community-wide need to establish a density forecast benchmark. To support this effort, a publicly available SET HASDM density *forecast* database is being developed. Given its use in U.S. Government operations, it may offer the research community a practical reference for evaluating and comparing other forecasting approaches.

#### NOAA SWPC WAM-IPE

WAM-IPE is a physics-based model developed by NOAA and University of Colorado Boulder researchers to provide operational specification of the ionosphere, thermosphere, and lower atmosphere conditions. The model captures the impact of solar and geomagnetic conditions as well as the perturbations from Earth’s lower atmosphere on the upper atmosphere. WAM-IPE is itself a coupled model consisting of two components, WAM and IPE. The WAM is a General Circulation Model (GCM) extending from the Earth’s surface to about 600 km altitude under average solar activity conditions; while the IPE (ionosphere-plasmasphere-electrodynamics) model provides the plasma component of the atmosphere, extending from 90 km to approximately 10,000 km [53, 54]. WAM treats major species O, O_2_, and N_2_ self-consistently, including large-scale transport, simple oxygen chemistry, and mutual diffusion both vertically and horizontally. The WAM lower atmosphere sources include thermospheric tides in the lower thermosphere dynamo region, changes in stratospheric circulation, molecular and atomic atmospheric composition, and composition variability. These mechanisms are one-way coupled to IPE to enable the plasma to respond to changes driven by the neutral atmosphere [55, 56]. Paths that allow the neutral thermosphere to respond to plasma forcing do not yet exist. WAM has a resolution of 1.89 degrees in latitude and longitude (94$\times$192) with 150 vertical levels ranging from the ground to 600 km altitude. The neutral density has been extended to 1000 km by incorporating MSIS helium above the WAM upper boundary. WAM-IPE uses an arithmetic mean of daily F10.7 and an 81 day average to feed a combination of the solar EUV irradiance model for aeronomic calculations (EUVAC) and high-resolution EUVAC (HEUVAC) models [57], while the solar wind-magnetospheric driving of the high-latitude electric field is calculated by the Weimer empirical model [50].

WAM-IPE has been operational at NOAA since July 2021. Two Concepts of Operations (ConOps), WAM-IPE Forecast System and WAM-IPE Real-time Nowcast System, are currently running in operation at NOAA. Different from all other models, the real-time portion of WAM-IPE utilizes real-time solar wind parameters every 5 min from NOAA’s spacecrafts located at (L1) while the forecast portion uses the forecast Kp from SWPC. These drivers serve as key inputs to the Weimer empirical model, which calculates the high-latitude electric fields required to model the impacts of geomagnetic activity on the system. WAM-IPE model has also been implemented in a couple of NSF and NASA HPCs.

Although the WAM-IPE model is not available for implementation on a system outside of NOAA, we think it is important to include it in this article because (1) the DoC is likely to utilize WAM-IPE or establish tools with WAM-IPE as one of its thermospheric density models in their Traffic Coordination System for Space (TraCSS) system, and (2) WAM-IPE’s global thermosphere density nowcast and forecast outputs are available operationally for integration into astrodynamics toolkits. Unlike HASDM, WAM-IPE does not currently employ data assimilation to correct the thermosphere; the current data assimilation scheme in WAM-IPE is only applied below 60 km altitude. It is important to note that WAM-IPE has not yet been validated to determine if it has attained the level of accuracy required for STM operations. SWPC continues to validate and improve the WAM-IPE model with plans to work with the larger space weather enterprise to incorporate data from satellites used in the COSMIC-2 and GOLD missions as well as other commercial satellite providers. This includes development of data assimilation and two-way coupling schemes for the Ionosphere-Thermosphere region.

WAM-IPE has been operational at NOAA since July 2021. The official operational output that has been archived from July 2021 to July 2022 is available through the SWPC-CCMC joint collaboration.^28^ More information about the model and operational outputs since 21 March 2023 can be found on the Amazon Web Services (AWS) WAM-IPE website.^29^ WAM-IPE version 1.1 was used to produce results in this work. WAM-IPE version 1.2 is now available, but only ranges back to July 2023.

## Analysis

This section provides various analyses comparing the operations-ready density models to each other, to HASDM and WAM-IPE, and to GRACE-FO and Swarm density products. For the purpose of this paper, only the most recent versions of the operations-ready density models are analyzed (i.e., DTM2020, JB2008, MSIS2.0, TIE-GCM 2.0). Because true global density is not available, operations-ready models are compared to HASDM density for global performance evaluation and the propagation analysis. April 2023 is the chosen time period for this study because WAM-IPE operational densities became available in March 2023, and this time period includes both quiet and storm conditions. Plots directly comparing HASDM and WAM-IPE are presented for the first time, as HASDM density data post-2020 are not yet publicly available. We also notably include a comparison of HASDM *forecast* density to WAM-IPE forecast density in Sect. 5.5; this is the first time HASDM *forecast* density has been publicly shared.

This section is not meant to be a comprehensive evaluation of each density model, but rather an example of model performance during quiet and geomagnetic storm conditions and moderate solar irradiance. One month of model assessment is not sufficient to determine overall model performance. Model biases fluctuate between time periods for a variety of reasons; a certain model that performs well during this particular time period of April 2023, may not perform well a month later, during another season, during another geomagnetic activity level, or during a different phase of the solar cycle.

### Global Mean Density

**Fig. 6:**
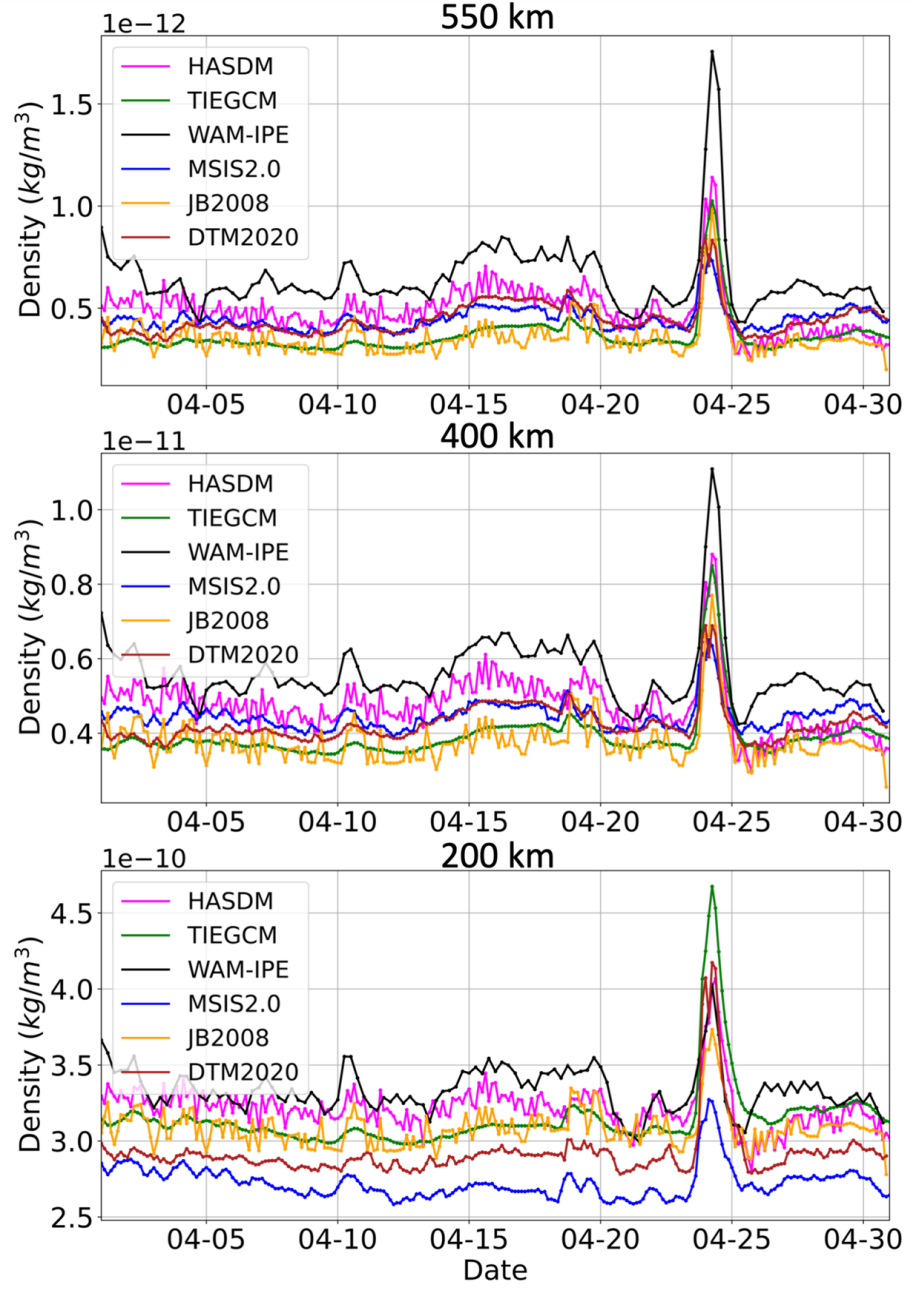
Globally averaged density at altitude shells of 200, 400, and 550 km

Figure 6 shows the April 2023 global mean density time-series of each model at altitude shells of 200, 400, and 550 km in the bottom, middle, and top plot, respectively. Overall, the density is relatively low during quiet conditions, before April 24th and after April 25th, and peaks during the geomagnetic storm on April 24th. In addition to the peak from the geomagnetic storm, there is a smaller density enhancement that occurs April 14–20 caused by an increase in solar irradiance (as shown in Fig. 3 by F10.7, S10, M10, and Y10). JB2008, HASDM, and WAM-IPE densities include more high frequency signals at approximately 10–20% amplitude levels which are largely absent in DTM, MSIS2.0, and TIE-GCM; at 200 km altitude all models except for TIE-GCM display this higher frequency (3–6 h) variability although it is still most pronounced in JB2008 and HASDM. The high frequency signals appear to be driven by model sensitivity to Kp/Ap in HASDM and JB2008. WAM-IPE global mean density is noticeably higher than other models at 400 and 550 km altitude during quiet and storm conditions. Although WAM-IPE is slightly higher than other models at 200 km altitude, this is only the case during quiet conditions; TIE-GCM has the highest density during the storm at 200 km altitude. JB2008 and TIE-GCM have similar day-to-day performance at all three altitude shells during this time period. Similarly, DTM and MSIS2.0 agree with each other at 400 and 550 km altitude, but are separated and lower than other models at 200 km. There is a predominantly positive correction applied to JB2008 in HASDM, which is solved for via assimilation of real-time satellite position data, showing that satellite data indicates higher density than JB2008 during quiet and storm conditions. HASDM and WAM-IPE densities appear to show post-storm overcooling (cooling to a lower level of density than the quiet conditions before the storm [58–60]. This indicates that the assimilated information from satellite orbits in HASDM shows post-storm cooling and suggests that WAM-IPE models post-storm constituent dynamics better than TIE-GCM. We note that solar activity is high enough during this time period such that TIE-GCM maximum altitude is higher than 550 km and thus does not need to be extrapolated to obtain these results. Overall, at the higher altitude of 550 km where most LEO satellites orbit, there is a factor of 2 difference between the model with the lowest (TIEGCM and JB2008) and highest (WAM-IPE) global density outputs. Excluding WAM-IPE at this altitude shell, that difference is approximately 30%. This large discrepancy in absolute global densities is an indication of the current status of thermospheric density modeling.

### Global Mean Density Errors Relative to HASDM

**Fig. 7:**
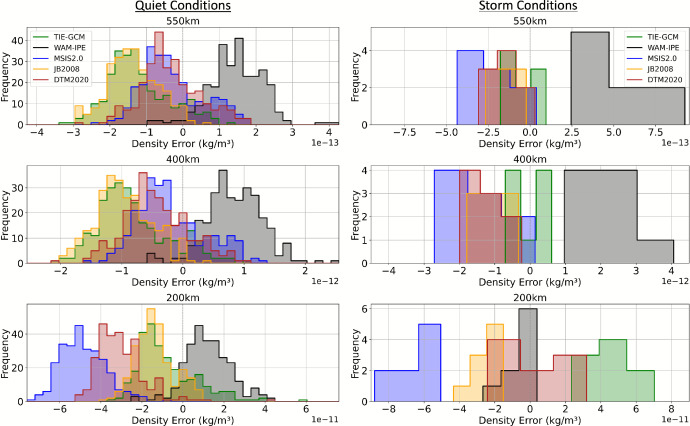
Histograms of global mean density error with respect to HASDM during quiet and storm conditions at altitude shells of 200, 400, and 550 km

Now we quantify the performance of each model’s density by comparing it to HASDM density. Figure 7 presents histograms of the global mean density error, computed for each model with respect to HASDM, at each altitude shell. Additional time series plots of mean density error metrics, including the mean, standard deviation, and RMSE with respect to HASDM, are provided in the supplementary material to support this analysis. Separate histograms are shown for quiet and storm conditions to highlight differences in model performance under varying geomagnetic conditions. The quiet conditions histogram is based on 29 days of data, while the storm conditions histogram represents a single day (April 24).

The most notable feature in both the quiet and storm conditions histograms is that WAM-IPE consistently exhibits a positive bias, indicating overprediction, across all altitude shells, with the exception of 200 km during storm conditions. Focusing now on quiet geomagnetic conditions, TIE-GCM and JB2008 demonstrate similar performance across all altitudes. Likewise, DTM2020 and MSIS2.0 show comparable behavior at 400 km and 550 km, with both models exhibiting mean biases closer to zero than JB2008 and TIE-GCM. However, at 200 km, this trend reverses: DTM2020 and MSIS2.0 display a larger negative bias than JB2008 and TIE-GCM, with MSIS2.0 showing the greatest underprediction. During storm conditions (April 24), the density model errors show more variability compared to quiet times, with broader distributions and more pronounced biases. WAM-IPE exhibits a strong positive bias across all altitudes, indicating significant overprediction relative to HASDM, particularly at 400 km and 550 km. At 200 km, however, WAM-IPE shifts toward a smaller positive bias, becoming more comparable to the other models. MSIS2.0 has the largest negative bias at all altitudes, particularly at 200 km. JB2008 and DTM2020 cluster more tightly between MSIS2.0 and TIE-GCM across altitudes, generally showing moderate underpredictions. These results underscore how geomagnetic disturbances affect each model differently and reinforce the importance of evaluating density model behavior under various geomagnetic and solar conditions.

### GRACE-FO and Swarm Density Comparison

This section provides a comparison of each model’s density to independent density data products from the GRACE-FO and Swarm missions during the month of April 2023. The GRACE-FO mission [61, 62] consists of a pair of satellites in circular, near-polar orbits, separated by approximately 240 km along their orbital tracks. The pair of satellites are in a non-Sun-Synchronous-Orbit (non-SSO) and therefore shift by one degree in local time each day. The initial mean altitude was about 510 km and decreased to 500 km in early 2023. With the main science objective of sensing variations in Earth’s gravitational potential, these satellites were launched in May 2018 to extend the data set of the original GRACE satellites [63], which flew 2002–2017. In addition to their primary objective, the satellites provide measurements of non-conservative forces from highly precise accelerometers that can be used to infer the neutral mass density of the thermosphere at high cadence (1 Hz) and resolution ($\sim$7.6 km along the orbit).

The density is obtained by inverting the x-component of the aerodynamic acceleration

3 $$\begin{aligned} \overrightarrow{a}_\textrm{aero} = \ \frac{1}{2}\rho \frac{\overrightarrow{C}_\textrm{aero}}{m}v_\textrm{rel}^{2} \end{aligned}$$

where $\rho$ is the neutral mass density, m is the satellite mass, ${\overrightarrow{v}}_{rel}$ is the satellite velocity vector relative to the atmosphere, and $\overrightarrow{C}_{aero}$ is the aerodynamic coefficient vector intrinsically scaled by the satellite’s cross-section area [64]. Equations 1 and 3 are related through the projection of the aerodynamic acceleration onto the direction of the relative velocity

4 $$\begin{aligned} \vec {a}_{\text {drag}} = \left( \vec {a}_{\text {aero}} \cdot \frac{\vec {v}_{\text {rel}}}{|\vec {v}_{\text {rel}}|} \right) \frac{\vec {v}_{\text {rel}}}{|\vec {v}_{\text {rel}}|} \end{aligned}$$

implying that

5 $$\begin{aligned} C_D A = \vec {C}_{\text {aero}} \cdot \frac{\vec {v}_{\text {rel}}}{|\vec {v}_{\text {rel}}|} \end{aligned}$$

where $\cdot$ denotes the dot product of two vectors. The aerodynamic acceleration is obtained by removing the modeled radiation pressure acceleration from the measured acceleration, which is calibrated using data from the GPS receiver. The density is derived from the x-component of the aerodynamic acceleration because the bias of this component can be accurately determined through precise orbit determination. In contrast, the acceleration due to drag could be affected by the less accurate y- and z-components of the bias [65]. Equation 3 indicates that the scale of the density observations depends on the aerodynamic coefficient vector, which is sensitive to the underlying gas-surface interaction model (GSI) [22]. The GRACE-FO density data were produced assuming diffuse reflections with incomplete accommodation as the GSI model, with an energy accommodation coefficient of 0.85. At 500 km altitude during solar minimum, this assumption may cause a scaling effect of 5% of the aerodynamic coefficient vector [17].

**Fig. 8:**
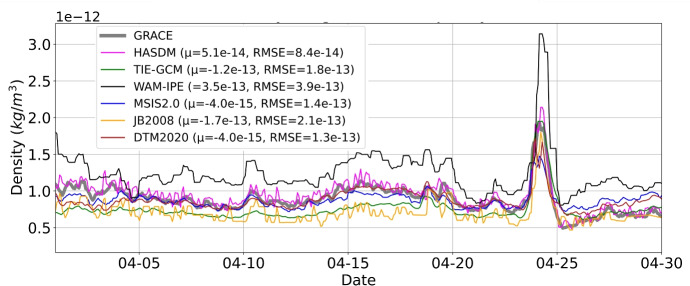
Orbit-average GRACE-FO and model density. Mean error and RMSE with respect to GRACE-FO are shown in the legend

Figure 8 provides the April 2023 orbit-average model and GRACE-FO accelerometer-derived densities. The mean error and RMS error (RMSE) of each model with respect to GRACE-FO are included in the plot legend. HASDM, DTM, and MSIS2.0 densities are most aligned with the GRACE-FO accelerometer-derived densities. WAM-IPE density is noticeably higher than other models and GRACE-FO. JB2008 and TIE-GCM have similar performance and have lower density than GRACE-FO. The GRACE-FO orbit-average accelerometer-derived densities show post-storm cooling, which is also captured by HASDM.

**Fig. 9:**
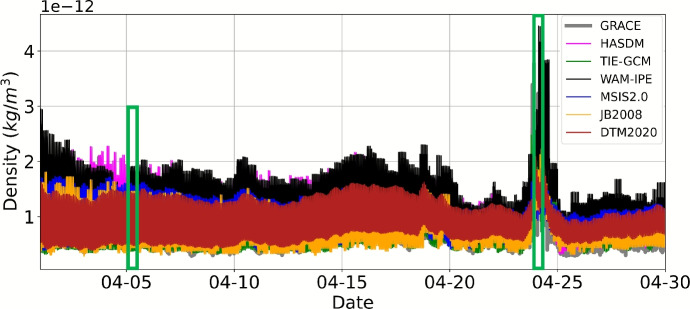
Model density along the GRACE-FO trajectory for month of April 2023

**Fig. 10:**
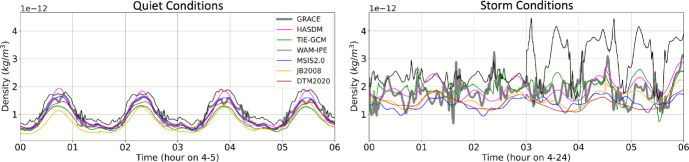
Model density along the GRACE-FO trajectory ($\sim$500 km altitude) during 12 h of quiet and storm conditions

Figure 9 provides the density along the GRACE-FO trajectory for each model (not orbit-average). We take a closer look at the quiet and storm conditions in Fig. 10, indicated by the green boxes in Fig. 9. All models have similar performance to GRACE-FO during quiet conditions. The correction between JB2008 and HASDM appears to bring the HASDM density much closer to the GRACE-FO accelerometer-derived density. GRACE-FO’s polar orbit traverses through midnight and local noon, and is located at the equator during local midnight. After the storm begins on April 24th at 3 Universal Time (UT), WAM-IPE density is higher than the other models and GRACE-FO. The storm time condition density behavior is more complicated; there are no clear density peaks as in the quiet conditions case. This is because the GRACE-FO orbit has shifted and is traversing through 10 and 22 LST (morning and evening) rather than near local noon and midnight, as it was during the quiet conditions. Interestingly, TIE-GCM and WAM-IPE, the physics-based models, are the only models that capture some of the high-rate variability of the GRACE-FO accelerometer-derived density, although TIE-GCM better matches the observed variability. The remaining models, all empirical-based, are only able to capture the average behavior of the true density via a relatively smooth sinusoid; this is likely due to the limited time resolution of the model drivers (3 h geomagnetic and daily solar flux indices).

The next independent comparison is performed using mass densities from the Swarm-B and Swarm-C satellites’ GPS accelerometry. Van den IJssel et al. [35] developed a method to determine thermospheric density along the tracks of three Swarm satellites (orbits ranging from 430 to 510 km altitude) using GPS tracking data. Their method was developed in response to finding several anomalies in the Swarm satellite mission accelerometer data. Thermospheric density at the satellite location is derived from non-gravitational accelerations based on precise orbit determination (POD) data only. These data are an official Level 2 data product, DNSxPOD, provided by ESA (see Data Availability Statement for more information on ESA Swarm data products). Both Swarm-B and Swarm-C are in nearly polar, non-SSO orbits. Swarm-B is higher in altitude (488–508 km) and slightly lower in inclination, while Swarm-C has an orbit altitude range of 447–471 km.

**Fig. 11:**
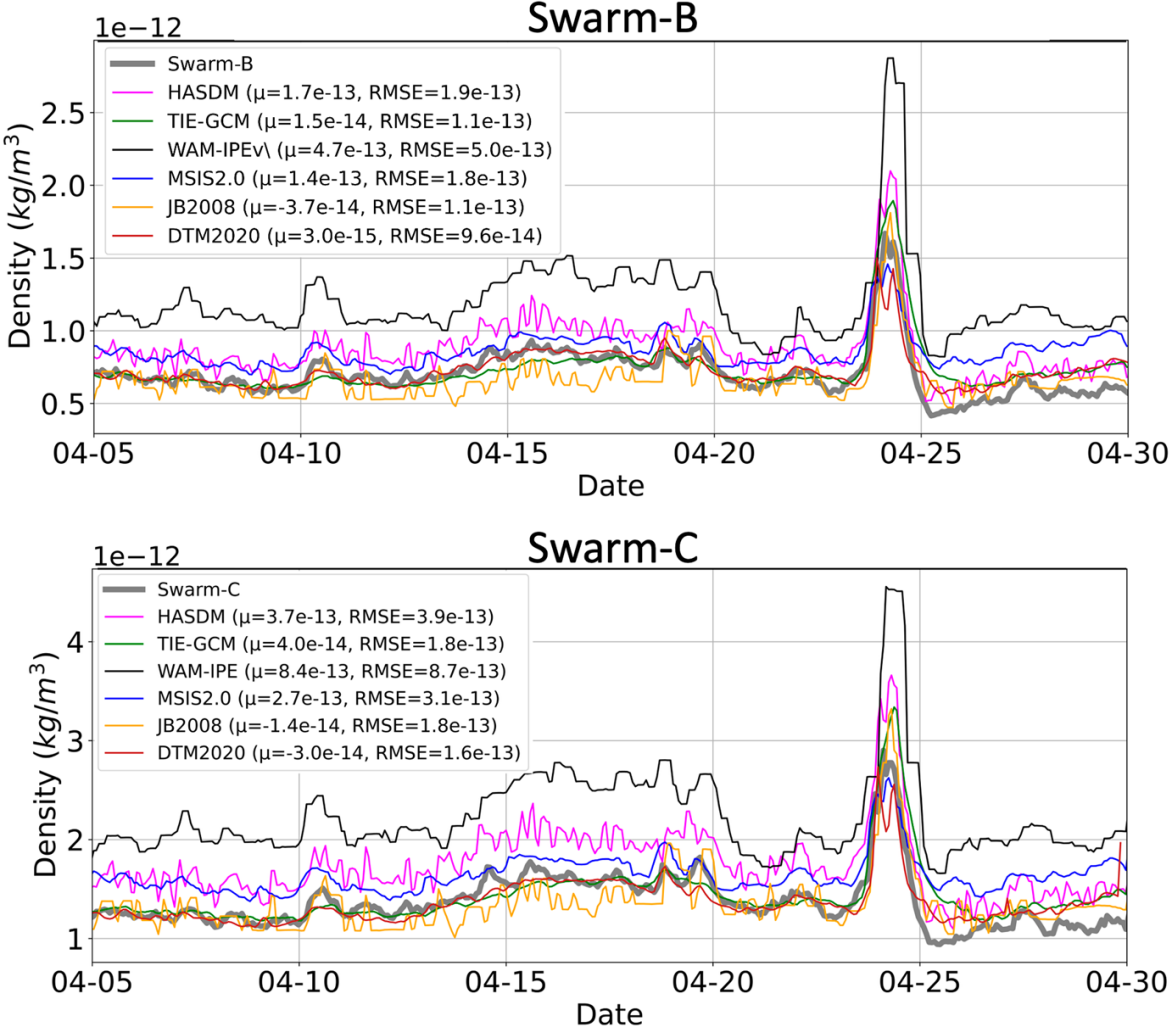
Orbit-average accelerometer-derived density and model density for Swarm-B and Swarm-C. Mean error and RMSE with respect to Swarm-B and Swarm-C are shown in the legend

Figure 11 provides the April 2023 orbit-average model densities and the Swarm-B and -C mission densities along the orbits of Swarm-B and -C. The mean error and RMSE of each model with respect to Swarm-B and Swarm-C are included in the plot legend. DTM, JB2008, and TIE-GCM densities are most aligned with the Swarm mission densities. WAM-IPE density is noticeably higher than other models and Swarm density. MSIS2.0 and HASDM have similar performance and have higher density than Swarm. The Swarm mission orbit-average densities show post-storm cooling, which is captured by HASDM.

**Fig. 12:**
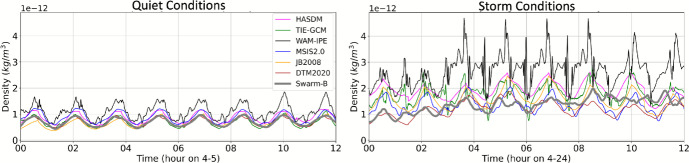
Model density along the Swarm-B trajectory ($\sim$500 km altitude) during 12 h of quiet and storm conditions

**Fig. 13:**
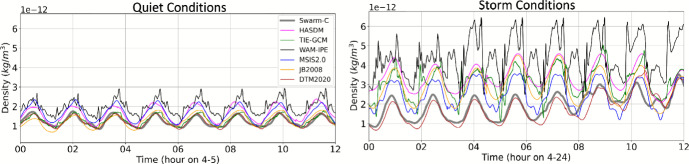
Model density along the Swarm-C trajectory ($\sim$460 km altitude) during 12 h of quiet and storm conditions

A 12 h example of quiet and storm conditions for Swarm-B and Swarm-C are provided in Figs. 12 and 13, respectively. Similar to the GRACE-FO comparison, all models have similar performance to Swarm during quiet conditions, but density behavior is more complicated during storm conditions. After the storm begins on April 24th at 3 Universal Time (UT), WAM-IPE density is higher than the other models and GRACE-FO. DTM2020 aligns noticeably well with Swarm-C density during storm conditions.

The ratio between the peak storm density and pre-storm quiet conditions density can be used as an indication of model performance. The pre-storm density is the mean density over the 2 days preceding the storm (April 21–23), while the peak storm density is the maximum density during the storm. Table 2 provides such ratios for each model (rows) compared to that of GRACE-FO (column 1), Swarm-B (column 2), and Swarm-C (column 3) density data products. The ratios observed by each satellite are shown in the first row. Each model peak-to-quiet density ratio is based on densities sampled at the locations of each satellite. Values provided in italics indicate that the model has similar behavior to the satellite mission density ratio (within 10%). This measure of performance is similar to evaluating model density that has been corrected for bias. The models that align best with the satellite mission density ratios are provided in italics. Both DTM and HASDM have a similar peak-to-quiet density ratio as the satellite mission density data for both Swarm satellites. While WAM-IPE has a similar peak-to-quiet density ratio as the satellite mission density for GRACE-FO. Analyzing model performance in this way reveals that DTM and HASDM have aligned performance to Swarm mission density data at both Swarm altitudes when model bias is corrected. The notable exception is alignment with GRACE-FO for this time, which is poor for almost all models. This can be a result of storm and/or quiet time errors at the particular local-times of the GRACE-FO orbit or a result of the higher spatial resolution in the GRACE-FO density product.

**Table 2:** Density ratio between the peak storm and pre-storm quiet conditions

Model / satellite	GRACE-FO	Swarm-B	Swarm-C
	(484–516 km)	(488–508 km)	(447–471 km)
Observed density ratio	**4**.**8**	**3**.**0**	**2**.**8**
DTM2020	2.6	*2.7*	*2.8*
HASDM	3.4	*3.1*	*2.7*
JB2008	3.5	3.9	3.4
MSIS2.0	2.2	2.5	2.2
TIE-GCM	4.0	4.3	3.7
WAM-IPE	*4.5*	4.9	3.4

Our findings align with those of Waldron et al. [66], who conducted a similar comparison of multiple thermospheric density models for a two-week period in November 2018. While their study leveraged ICESat-2 data and included the CTIPe model, the general conclusion was consistent with ours: no single model consistently outperforms the others across all conditions. We do not include CTIPe here, as it does not currently meet our criteria for an operations-ready model. Our work extends the existing body of research by focusing on a different time period (April 2023), incorporating the WAM-IPE model, and emphasizing operational readiness for real-time use. Together, these studies highlight the continued need for calibrated, operationally viable density models that perform reliably across a range of space weather conditions.

### Propagation Analysis

This simulation scenario is created to study the in-track position difference between propagating a nominal 3U CubeSat through a density field generated with each operations-ready density model and with HASDM density. Orekit [67] is used to model the orbit dynamics. For this study, a simplified dynamical model was applied, consisting of only a zero-degree, zero-order spherical Earth gravity field and atmospheric drag. We augmented the standard Orekit atmospheric drag model to utilize the density output from each of the thermospheric density models evaluated in this study, rather than one of the conventional underlying density models included with Orekit, such as Harris Priester. For the purpose of this study, HASDM global density is considered true density. A simulated satellite in a circular orbit is propagated with each density model for 1 day at two altitude shells: 400 km and 550 km. All propagations are initialized with the same ephemeris initial conditions (i.e., position and velocity). Then the position difference between each model’s propagation and the HASDM-based propagation at the end of each day is computed. Because there is no baseline error in the simulation, any deviation in the propagated position can be attributed to differences in the modeled density. A smaller position difference therefore indicates a density model that is better aligned with HASDM. This analysis is repeated for each day throughout April 2023.

A bias correction is applied to each model density for propagation. To obtain the corrected model density, the mean ratio between HASDM density and the model density is computed for the 3 days leading up to but not including the propagation day (information available in operational environment). The density ratio multiplies the model density to provide a bias correction. This approach effectively emulates estimating the satellite ballistic coefficient in an operational orbit determination filter to remove bias.

Each propagation is initialized with the same ephemeris initial conditions. The final in-track position difference between the propagation with each density model and the propagation with HASDM is then computed. A smaller in-track position difference indicates a better-calibrated density model relative to HASDM. Each trajectory is propagated using the same dynamics; the only difference is the underlying density model.

**Fig. 14:**
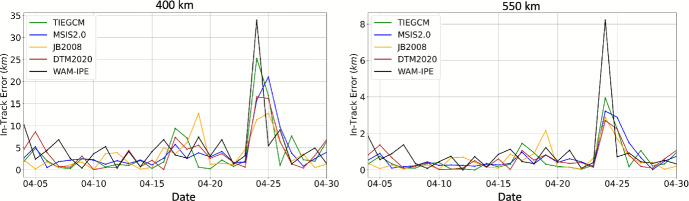
Absolute in-track position error at 400 km (left) and 550 km (right) for a 1 day orbit propagation using model density (with a bias correction applied) with respect to a propagation using HASDM density

Figure 14 provides the in-track position difference between the 1 day propagation with each density model with respect to HASDM for each day in April 2023. The plot begins on April 4th due to the 3 day bias calculation. Overall, the in-track position error is higher at 400 km altitude compared to 550 km due to the exponential decay of density, and therefore also drag, as a function of altitude. All models lead to in-track positions that are within 12 km (2 km) of the HASDM propagation at 400 km (550 km) during quiet conditions. As expected, the bias correction is less effective during the storm, causing a higher in-track error for all models. WAM-IPE has a noticeably higher in-track error than other models during storm conditions, especially at 550 km altitude. For additional context, the same orbit propagations were performed with atmospheric drag turned off, representing a case in which no density model was applied. The resulting in-track position errors averaged 85 km during quiet conditions and 125 km during storm conditions at 400 km altitude, and 9 km and 15 km, respectively, at 550 km altitude. These cases are not shown in the plots, as including them would significantly distort the axis scales.

### HASDM and WAM-IPE Forecast Density

**Fig. 15:**
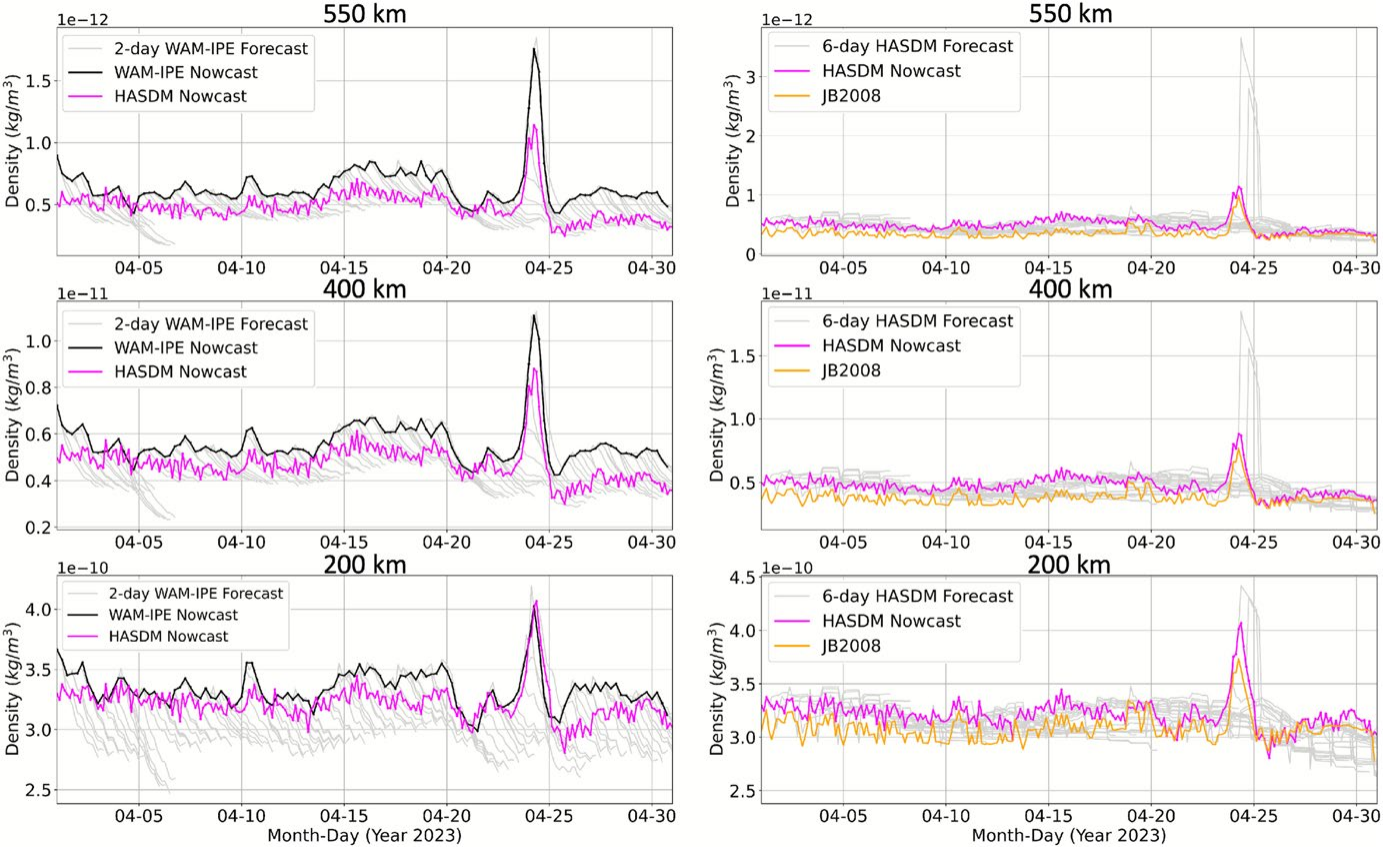
Globally averaged WAM-IPE 2 day forecast (left) and HASDM 6 day forecast (right) compared to nowcasts from WAM-IPE, HASDM, and JB2008

Similar to the HASDM nowcast, the April 2023 HASDM 6 day forecast solution was also obtained for the purpose of this work, as the HASDM forecast density data are not publicly available. Figure 15 shows a) the WAM-IPE nowcast (black) and 2 day forecast (grey) densities with HASDM nowcast density (magenta) overlayed for context, and b) the HASDM nowcast (magenta) and 6 day forecast (grey) densities with JB2008 (orange) for context. The HASDM forecast density is produced using the inherent HASDM forecast algorithm in combination with the SET operational solar and geomagnetic forecast indices (S10, M10, Y10, F10, Ap, and Dst); while the WAM-IPE forecast density is produced using the inherent WAM-IPE forecast algorithm in combination with the NOAA SWPC forecast indices (F10 and Ap/Kp). The global mean density at altitude shells 200 km, 400 km, and 550 km is shown with a 3 h resolution. The 6 day HASDM forecast density generally tracks the nowcast during quiet conditions but has apparent performance degradation around storm time. During the April 2023 storm conditions, HASDM produces a false negative prediction of the storm and then overpredicts the storm after onset. This performance degradation is related to the challenges of solar and geomagnetic forecasting. The WAM-IPE 2 day forecasts (grey) trend downward with forecast time, relative to the nowcast, to values that are nearly always incorrect and too low. Overall, the HASDM and WAM-IPE operational density forecast performance in April 2023 demonstrates the need for continued research and development to improve density forecasting methods for benefit of satellite operations.

## Conclusion

This article provides a description of each operations-ready density model, which we define as a model that is well-documented, has verified and quantified model performance, and provides publicly available model code for implementation on a user’s own system. A performance analysis of the operations-ready density models, with the addition of HASDM and WAM-IPE, is presented. This analysis is meant to provide an example of model performance over a limited time period rather than being a comprehensive evaluation of each model. The global mean density plots and comparison to GRACE-FO and Swarm mission densities evaluate each model’s performance independent of HASDM. In contrast, the density error histograms and propagation analysis are computed relative to HASDM, as it is the closest to global true density that is available.

*Global Density Evaluation:* The global mean density plots show that WAM-IPE has distinctly higher density at 200, 400, and 550 km altitude shells throughout the month of April 2023. JB2008 and TIE-GCM have similar global mean densities, as do DTM and MSIS2.0. Global density error histograms with respect to HASDM showed that WAM-IPE has a distinctly higher bias than other models, which aligns with the findings in the global mean density plots.

*Model Bias*: JB2008 and TIE-GCM are similar to each other in terms of bias during the time period analyzed, as are DTM and MSIS2.0 at 400 and 550 km altitude. At 200 km altitude, MSIS2.0 has a distinctly higher RMSe than other models. At 550 km altitude, MSIS2.0 and DTM are the better performing models in terms of bias and RMSe, followed by TIE-GCM and JB2008, then WAM-IPE. Orbit-averaged HASDM, DTM, and MSIS2.0 align best with GRACE-FO accelerometer-derived density.

*Variability:* The physics-based models, TIE-GCM and WAM-IPE, capture some of the local variability of the GRACE-FO density, while the empirical-based models are only able to capture the average behavior of the GRACE-FO density via a smooth sinusoid during disturbed conditions in the morning and night. DTM, JB2008, and TIE-GCM densities are most aligned with the Swarm mission densities. The Swarm mission orbit-average densities show post-storm cooling, which is captured by HASDM. DTM2020 aligns noticeably well with Swarm-C density during storm conditions. JB2008 tends to model lower densities compared to GRACE-FO, Swarm, and most other models throughout the analyses; this is likely due to not enough thermosphere heating input to the model via model driver S10. An updated S10 index is expected late 2026 that will improve thermosphere heating in JB2008.

*Propagation Evaluation:* A propagation analysis is also presented in which a CubeSat is propagated with each model and compared to the HASDM propagation. Each model density has a bias correction applied before propagation, which effectively emulates estimating the satellite ballistic coefficient in an operational orbit determination filter to remove bias. All models have comparable performance during quiet conditions, while WAM-IPE has a noticeably higher in-track error than other models during storm conditions, especially at 550 km altitude.

*Forecast:* Lastly, the HASDM 6 day forecast density is revealed publicly for the first time and compared to the WAM-IPE 2 day forecast density. HASDM forecast density generally tracks the nowcast during quiet conditions but exhibits altitude-dependent performance degradation around storm time. WAM-IPE 2 day forecasts (grey) decay with time to values that are nearly always incorrect and too low.

*Future Work:* Overall, the analysis shows that any of the operations-ready density models (DTM2020, JB2008, MSIS2.0, TIE-GCM) are a viable option for satellite operations. A more comprehensive analysis is required to understand performance differences under the full range of solar and geomagnetic conditions. Results also demonstrate the need for continued research and development to improve density nowcasting and forecasting methods. SET is currently developing an improved nowcast S10 and forecast S10 and Dst, which should be available operationally in 2026. It is expected that an improvement to these indices will lead to improved JB2008 and HASDM performance. The WAM-IPE developers at NOAA SWPC have been investigating the overestimation of neutral density in the real-time portion and the reduction of density in the forecast. It has been confirmed that the overestimation of the density is directly related to the high-cadence solar wind drivers used in the model. None of the other models in this study utilize 5 minute solar wind drivers as their inputs. Using the high-cadence drivers can help the physics-based model capture the rapid changes in energy pulses that go into the system. However, the temporal variation of these external sources and consequence of response time of the neutral atmosphere leads to a much higher global density in the nowcast model results. The WAM-IPE team is working on a method to balance these drivers in order to level up the forecast results with respect to the nowcast while retaining the capability of rapid changes in the system associated with the external drivers. NOAA is also actively exploring real-time datasets and developing data assimilation and two-way coupling schemes for WAM-IPE to reduce current model bias.

In addition to density model-developer efforts, operational users have options for improving density/drag model performance. The first, simpler option is implementing ballistic coefficient estimation in the orbit determination filter to effectively remove density model bias. The effect of this approach is demonstrated in the Propagation Analysis section. Another, more sophisticated option is to develop an in-house data assimilation method to correct a chosen background density model using real-time local density measurements. Performance of such a data assimilation approach is dependent on several factors, including the quality, quantity and spatial distribution of measurements as well as the performance of the background density model.

For additional model comparisons, we recommend the NASA CCMC Comprehensive Assessment of Models and Events based on Library tools (CAMEL) thermosphere neutral density score card. Although currently under development, it will soon provide comprehensive model assessments of all operations-ready density models (plus a few more) during several historical storm periods.^30^

## Supplementary Information

 Additional time series plots of mean density error metrics, including the mean, standard deviation, and RMSE with respect to HASDM, are provided in the supplementary material to support and complement the analysis presented in the Sect. 5.2.

## Supplementary Information

Below is the link to the electronic supplementary material.

Supplementary file 1 (PDF 5101 KB)

## Data Availability

HASDM density data can be retrieved via SET’s website https://spacewx.com/hasdm/. GRACE-FO accelerometer density https://swarm-diss.eo.esa.int/#swarm%2FMultimission and Swarm density data https://swarm-diss.eo.esa.int/#swarm/Level2daily/Entire_mission_data/ are available from ESA as a Level 2 data product of the respective missions. TIEGCM is developed and maintained by the National Center for Atmospheric Research’s High Altitude Observatory (HAO) [55].

## Footnotes

1 https://www.gfz-potsdam.de/en/section/geomagnetism/data-products-services/geomagnetic-kp-index

2 https://www.ngdc.noaa.gov/stp/geomag/kp_ap.html

3 https://www.gfz-potsdam.de/en/section/geomagnetism/data-products-services/geomagnetic-kp-index

4 https://kp.gfz.de/en/hp30-hp60

5 https://kp.gfz.de/en/

6 https://www.swpc.noaa.gov/products/3-day-geomagnetic-forecast

7 https://www.swpc.noaa.gov/products/usaf-45-day-ap-and-f107cm-flux-forecast

8 https://celestrak.org/SpaceData/SpaceWx-format.php

9 https://wdc.kugi.kyoto-u.ac.jp/dst_realtime/index.html

10 https://spaceweather.cls.fr/services/radioflux/

11 https://swx-trec.com/vector/

12 https://space-geodesy.nasa.gov/techniques/tools/GEODYN/GEODYN.html

13 https://www.orekit.org/static/apidocs/org/orekit/models/earth/

14 https://www.ansys.com/products/missions/ansys-odtk

15 https://swami-h2020-eu.github.io/mcm/dtm2020.html

16 https://sol.spacenvironment.net/JB2008/

17 https://www.orekit.org/static/apidocs/org/orekit/models/earth/

18 https://www.mathworks.com/matlabcentral/fileexchange/56163-jacchia-bowman-atmospheric-density-model

19 https://map.nrl.navy.mil/map/pub/nrl/NRLMSIS/NRLMSIS2.0/

20 https://pypi.org/project/pymsis/

21 https://www.orekit.org/static/apidocs/org/orekit/models/earth/

22 https://space-geodesy.nasa.gov/techniques/tools/GEODYN/GEODYN.html

23 https://software.nasa.gov/software/GSC-17177-1

24 https://www.hao.ucar.edu/modeling/tgcm/tie.php

25 https://gallery.ecr.aws/swx-trec/tiegcm

26 https://spacewx.com/hasdm/

27 https://spacewx.com/hasdm/

28 https://ccmc.gsfc.nasa.gov/news/wamipe-update/

29 https://registry.opendata.aws/noaa-nws-wam-ipe/

30 https://webserver1.ccmc.gsfc.nasa.gov/camel/NeutralDensity/
