# Supplementary material for: A Survey of Current Operations-Ready Thermospheric Density Models for Drag Modeling in LEO Operations
Source: J Astronaut Sci. 2026 Mar 17;73(2):32. doi: 10.1007/s40295-025-00558-8 (PMC12995994; doi:10.1007/s40295-025-00558-8)
Supplement: Supplementary file 1 — (PDF 5101 KB) [file 40295_2025_558_MOESM1_ESM.pdf]

Supplementary Material for A Survey of Current Operations-Ready Thermospheric Density Models for Drag Modeling in LEO Operations

Shaylah Mutschler[1*], Marcin Pilinski[2], Sean Bruinsma[3], Eric Sutton[4], W. Kent Tobiska[1], Delores Knipp[5], Tzu-Wei Fang[6], Steve Casali[7], Vishnuu Mallik[8], Brandon DiLorenzo[1], Christian Siemes[9]

[1*]Space Environment Technologies (SET), Street, Pacific Palisades, CA, USA.
[2]Laboratory for Atmospheric and Space Physics (LASP), University of Colorado at Boulder, Boulder, CO, USA.
[3]Space Geodesy Office, Centre National d'Etudes Spatiales (CNES), Toulouse, France.
[4]Space Weather Technology, Research and Eductation Center (SWx TREC), University of Colorado at Boulder, Boulder, CO, USA.
[5]Smead Aerospace Engineering Sciences Department, University of Colorado at Boulder, Boulder, CO, USA.
[6]National Oceanic & Atmospheric Association (NOAA), Boulder, CO, USA.
[7]Omitron, Beltsville, MD, USA.
[8]Planet Labs PBC, San Francisco, CA, USA.
[9]Delft University of Technology, Delft, Netherlands.

*Corresponding author(s). E-mail(s): smutschler@spacewx.com;

1 Global Mean Evaluation Metrics

Here we quantify the performance of each model's density by comparing it to HASDM density via the metrics presented in (Sutton, 2018), (Sutton et al., 2021). These metrics include $\mu\left(\frac{m}{o}\right)$, $\sigma\left(\frac{m}{o}\right)$, and R$MSe\left(\frac{m}{o}\right)$, all in log space

$$\mu\left(\frac{m}{o}\right) = exp\left(\frac{1}{N}\sum_{i=1}^{N}\ln\frac{\rho_{m,i}}{\rho_{o,i}}\right) \tag{1}$$

$$\sigma\left(\frac{m}{o}\right) = \sqrt{\frac{1}{N}\sum_{i=1}^{N}\left(\ln\frac{\rho_{m,i}}{\rho_{o,i}} - \ln\mu\left(\frac{m}{o}\right)\right)^2} \tag{2}$$

$$RMSe\left(\frac{m}{o}\right) = \sqrt{\frac{1}{N}\sum_{i=1}^{N}\left(\ln\frac{\rho_{m,i}}{\rho_{o,i}}\right)^2} \tag{3}$$

where "m" is the *model* density and "o" is the *observed* density, or the closest to truth density that is available (i.e., HASDM). N is the total number of data points included in the calculation. For this work, the metrics are calculated globally for each altitude shell separately; so, N = 456, which is a data point every one hour in LST and every 10-deg in latitude (24 LSTs x 19 latitudes). The $\sigma\left(\frac{m}{o}\right)$ and $RMSe\left(\frac{m}{o}\right)$ quantities are best interpreted as percentages, using:

$$\% = 100 \times \left(\exp\sigma\left(\frac{m}{o}\right) - 1\right) \tag{4}$$

μ indicates density bias, where $\mu = 1$ is no bias between the model and HASDM. Root Mean Squared error (RMSe) is a good indicator of total model errors, and σ is an indication of the spread of the errors (i.e., variance). It is noted that an operational user of a density model can incorporate satellite ballistic coefficient estimation in their orbit determination filter to effectively remove the recently observed density model bias and consequently also reduce RMSe.

The global density evaluation metrics are provided for each model throughout the month of April 2023 in Figures 1-3 for altitude shells 200, 400, and 550 km. Metrics are computed between each model (m) and HASDM (o), globally, at a 3-hour time resolution. These metrics are a representation of the model performance across the globe, as opposed to a global-average, because they are computed on a point-by-point basis using the spatial resolution of HASDM, 10-deg latitude and 1-hour Local Sidereal Time (LST) (i.e., 10-deg longitude). In other words, the metrics in Figure 7 represent the spatial error against HASDM while using a consistent spatial resolution among all models. The inherent resolution of JB2008, as well as that of the HASDM corrections applied to it, is lower than what is required to capture certain density features. These features will manifest as "errors" even though they are actually additional signal (correct or not). Additionally, there is possible bias in HASDM density caused by the C_D applied to each calibration object during processing. The overall bias, RMSe, and variance between the models and HASDM increases as altitude increases; they are lowest at 200 km altitude (μ is closer to 1) and highest at 550 km altitude. During quiet times, WAM-IPE density has the largest positive bias ($\mu > \mathbf{1}$), whereas other models

have lower density than HASDM, indicated by $\mu < \mathbf{1}$. JB2008 and TIE-GCM have similar bias at all three altitude shells; DTM and MSIS have similar bias at 400 and 550 km altitude but differ at 200 km altitude. Each model's performance is distinct at 200 km altitude; the total global error, indicated by RMSe, is lowest for JB2008 and highest for MSIS2.0 at 200 km altitude. At higher altitudes, MSIS2.0 and DTM are the better performing models in terms of bias and RMSe, followed by TIE-GCM and JB2008, then WAM-IPE.

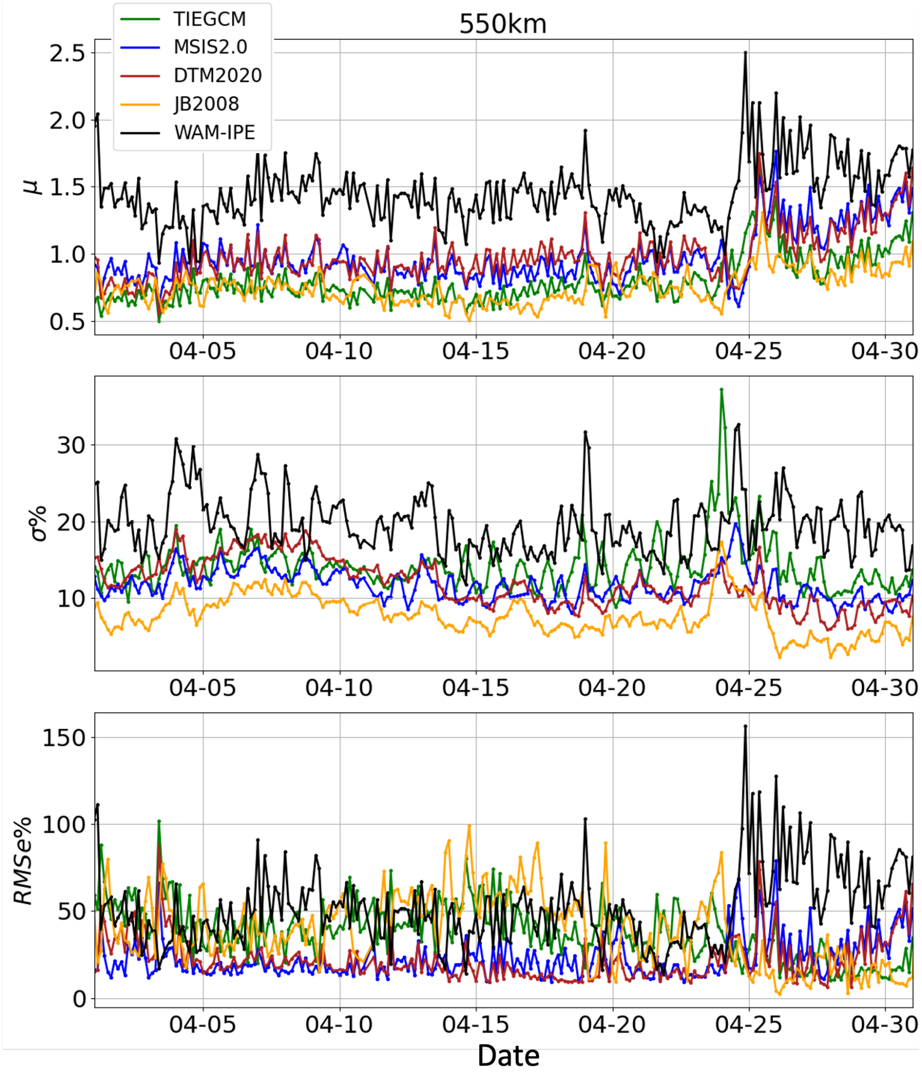

Fig. 1 Density evaluation metrics for each model with respect to HASDM at 550 km

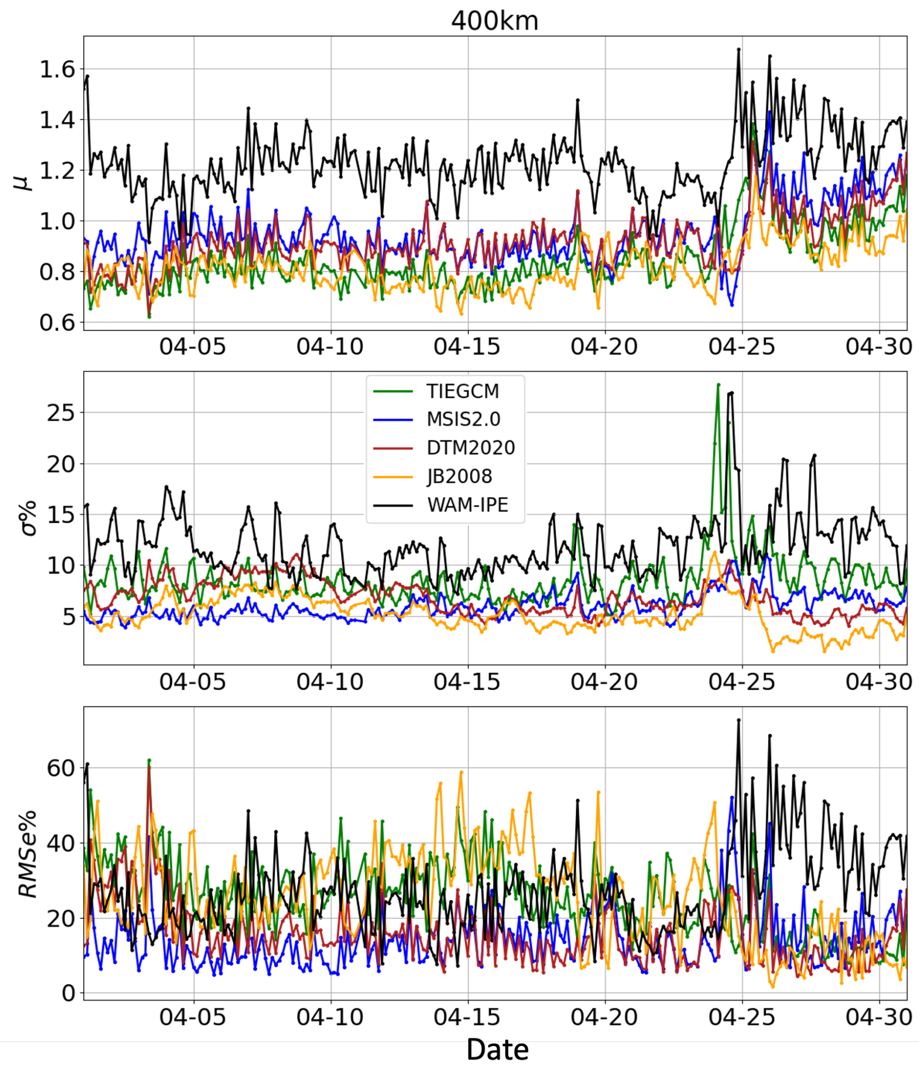

Fig. 2 Density evaluation metrics for each model with respect to HASDM at 400 km

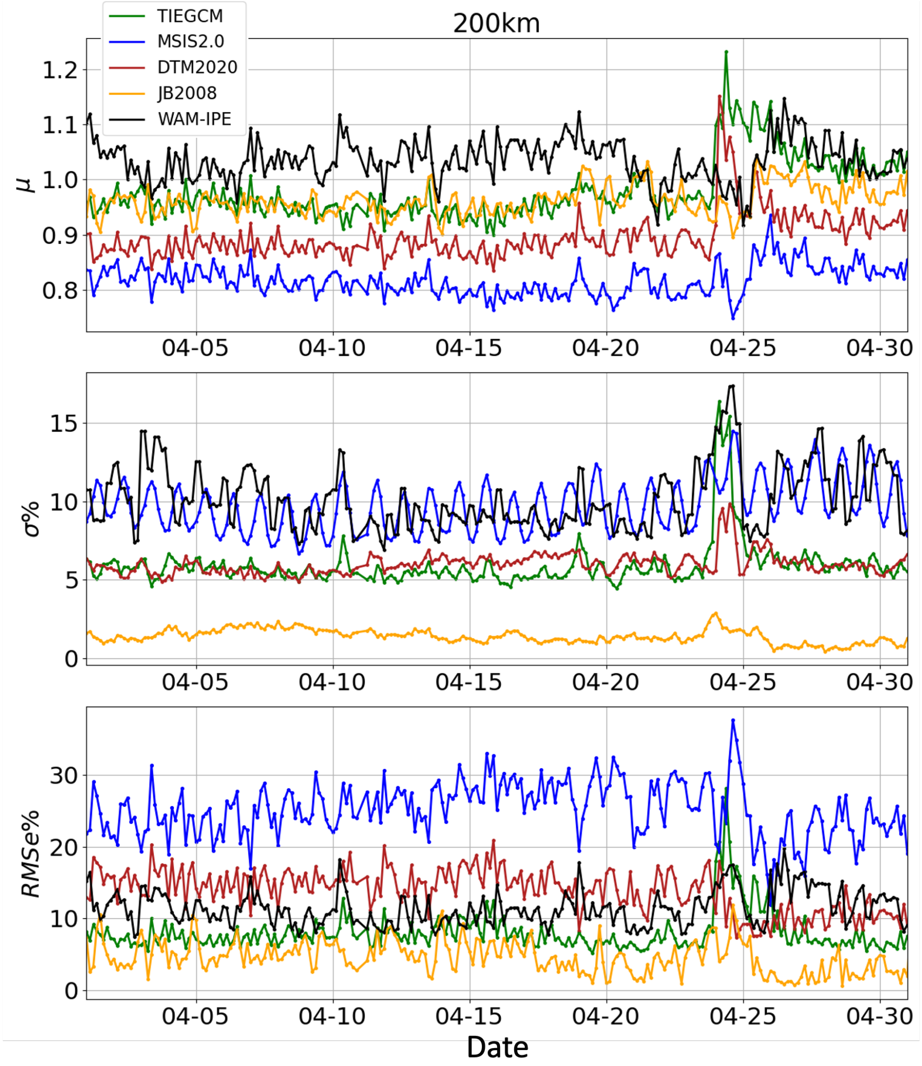

Fig. 3 Density evaluation metrics for each model with respect to HASDM at 200 km

References

Sutton, E., 2018. A new method of physics-based data assimilation for the quiet and distrubed thermosphere. *Space Weather,* pp. 736-753. DOI: 10.1002/2017SW001785

Sutton, E., Thayer, J.P., Pilinski, M., Mutschler, S., Berger, T., Nguyen, V., & Masters, D., 2021. Toward accurate physics-based specifications of neutral density using GNSS-enabled small satellites. *Space Weather.* DOI: 10.1029/2021SW002736
